# Carbohydrate Modified Diet & Insulin Sensitizers Reduce Body Weight & Modulate Metabolic Syndrome Measures in EMPOWIR (Enhance the Metabolic Profile of Women with Insulin Resistance): A Randomized Trial of Normoglycemic Women with Midlife Weight Gain

**DOI:** 10.1371/journal.pone.0108264

**Published:** 2014-09-26

**Authors:** Harriette R. Mogul, Ruth Freeman, Khoa Nguyen, Michael Frey, Lee-Ann Klein, Sheila Jozak, Karen Tanenbaum

**Affiliations:** 1 Department of Medicine, Division of Endocrinology, New York Medical College, Valhalla, New York, United States of America; 2 Departments of Medicine and Obstetrics and Gynecology, Albert Einstein College of Medicine, Bronx, New York, United States of America; Indiana University Richard M. Fairbanks School of Public Health, United States of America

## Abstract

**Rationale:**

Progressive midlife weight gain is associated with multiple adverse health outcomes and may represent an early manifestation of insulin resistance in a distinct subset of women. Emerging data implicate hyperinsulinema as a proximate cause of weight gain and support strategies that attenuate insulin secretion.

**Objective:**

To assess a previously reported novel hypocaloric carbohydrate modified diet alone (D), and in combination with metformin (M) and metformin plus low-dose rosiglitazone (MR), in diverse women with midlife weight gain (defined as >20lbs since the twenties), normal glucose tolerance, and hyperinsulinemia.

**Participants:**

46 women, mean age 46.6±1.0, BMI 30.5±0.04 kg/m^2^, 54.5% white, 22.7% black, 15.9% Hispanic, at 2 university medical centers.

**Methods:**

A dietary intervention designed to reduce insulin excursions was implemented in 4 weekly nutritional group workshops prior to randomization.

**Main Outcome Measure:**

Change in 6-month fasting insulin. Pre-specified secondary outcomes were changes in body weight, HOMA**-**IR, metabolic syndrome (MS) measures, leptin, and adiponectin.

**Results:**

Six-month fasting insulin declined significantly in the M group: 12.5 to 8.0 µU/ml, *p* = .026. Mean 6-month weight decreased significantly and comparably in D, M, and MR groups: 4.7, 5.4, and 5.5% (*p’s*.049, .002, and.032). HOMA–IR decreased in M and MR groups (2.5 to 1.6 and 1.9 to 1.3, *p’s = *.054, .013). Additional improvement in MS measures included reduced waist circumference in D and MR groups and increased HDL in the D and M groups. Notably, mean fasting leptin did not decline in a subset of subjects with weight loss (26.15±2.01 ng/ml to 25.99±2.61 ng/ml, *p* = .907. Adiponectin increased significantly in the MR group (11.1±1.0 to 18.5±7.4, *p*<.001) Study medications were well tolerated.

**Conclusions:**

These findings suggest that EMPOWIR’s easily implemented dietary interventions, alone and in combination with pharmacotherapies that target hyperinsulinemia, merit additional investigation in larger, long-term studies.

**Trial Registration:**

ClinicalTrials.gov NCT00618072

## Introduction

Progressive weight gain in the fourth and fifth decade**,** commonly reported by women in all ethnic and socio-economic groups [1], has profound health implications. As first demonstrated by the Nurses’ Health Study, weight gain of 10 kg (22 pounds) or more after the age of 18 is associated with development of Type 2 diabetes (T2DM) [2], [3] in women, and after adjustment for relevant covariates, has a linear effect on age-adjusted cardiovascular [4], [5] and all-cause mortality [6]. Moreover, excess adiposity negatively impacts job attainment, pay-scale, and marital status [7] with a greater economic and social burden on women. Thus, midlife weight gain, long regarded an inevitable consequence of aging, is a harbinger of diabetes, and multiple additional adverse health and psychosocial outcomes. Accordingly, interventions that reverse progressive midlife weight gain are critically needed to diminish the risks of diabetes and cardiovascular disease and improve the health status of women.

We previously reported that intractable midlife weight gain represented an early clinical manifestation of insulin resistance in a distinct subpopulation of healthy-appearing women – demarcated by increased glucose-mediated insulin response curves in the presence of completely normal glucose tolerance tests [8], [9]. We termed this disorder Syndrome W – to highlight its defining triad of **w**eight gain, **w**aist gain, and **w**hite-coat hypertension in **w**omen, and its presumed role as an alphabetic and a chronologic antecedent to Syndrome X, now known as metabolic Syndrome (MS). We proposed that, as in other well-defined disorders of insulin action in younger women – polycystic ovarian syndrome (PCOS) [10], premature adrenarche [11], and precocious puberty [12] – Syndrome W was a precursor of metabolic syndrome and diabetes at an apparently early, optimal stage for intervention.

Data from our initial pilot studies demonstrated that metformin (M) in combination with a novel hypocaloric carbohydrate modified diet (CMD) produced significant and sustainable weight loss in women with Syndrome W, with notable reductions in fasting insulin [13]. Additional 2–4 year follow-up in an intention-to-treat analysis of consecutive women who lost ≥10% of their body weight after one year of the treatment regimen, further suggested that the composite intervention prevented (NHLBI-defined) weight regain (≤2 kg) and the onset of overt glucose impairment [14]. These collective findings were consistent with initial observations in children that insulin elevations could be a cause as well as a consequence of weight gain [15] and an emerging consensus implicating hyperinsulinemia as a “proximate” or “root cause of insulin resistance and diabetes” [16]–[18], now increasingly documented by clinical [19], [20] and laboratory studies [21]–[23].

EMPOWIR (**E**nhance the **M**etabolic **P**rofile of **W**omen with **I**nsulin **R**esistance) was a pilot study to evaluate the previously reported dietary intervention and metformin treatment in the context of a placebo controlled, double blind, randomized clinical trial. The study objective was to determine whether CMD alone, or in combination with metformin (M) or metformin plus low-dose rosiglitazone (MR), could reduce insulin levels and improve established risk factors for diabetes and metabolic syndrome in women with Syndrome W. The study was designed to evaluate two hypotheses: (1) A significant proportion of women with progressive midlife weight gain have undetected and potentially correctable insulin elevations which impede weight loss. (2) Identification of hyperinsulinemia prior to the onset of overt glucose abnormalities and initiation of targeted dietary and pharmacologic interventions could reverse weight gain in midlife women and provide possible new treatment models for other comparable high risk populations.

## Subjects and Methods

### Design Overview

**Figure 1:**
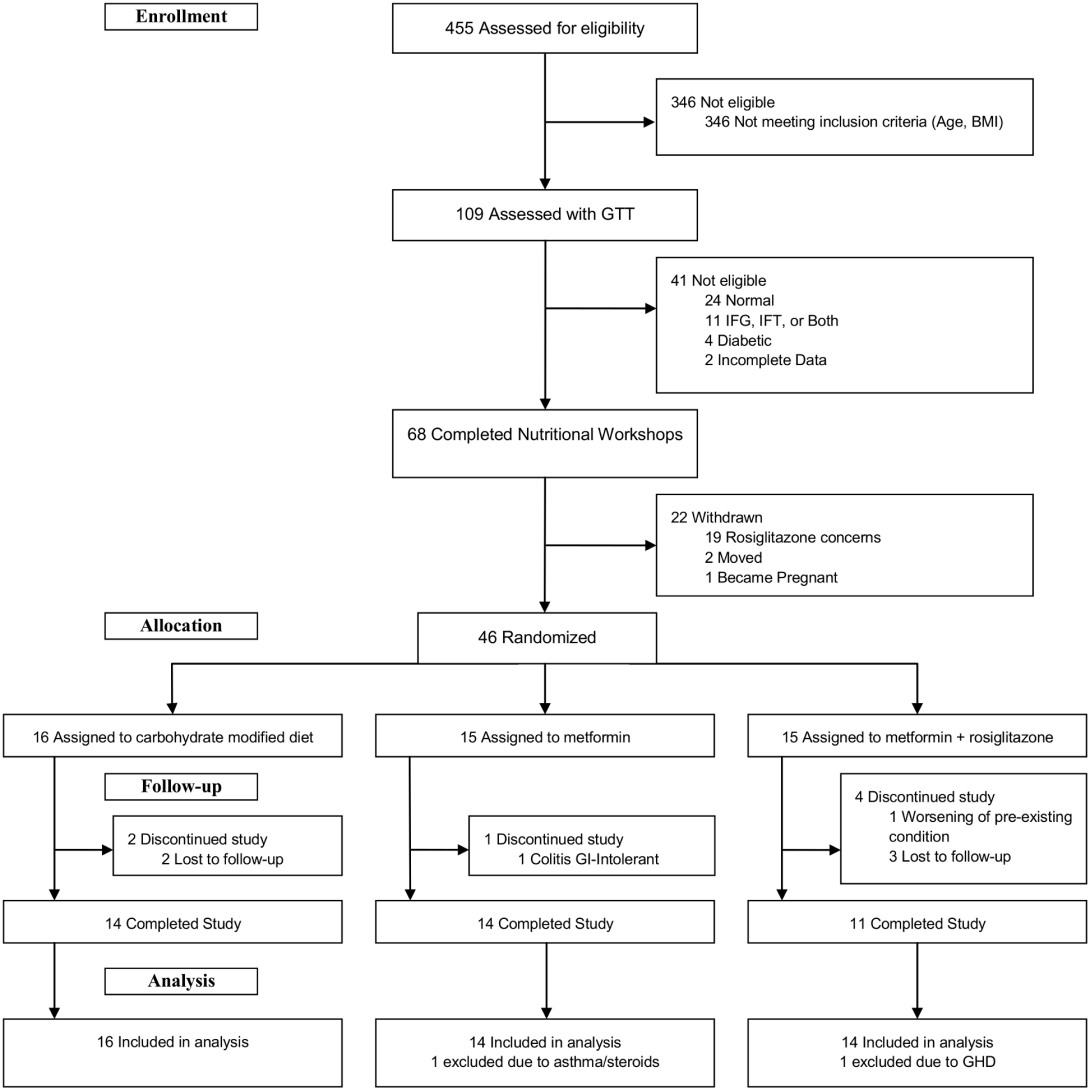
CONSORT Diagram.

EMPOWIR was a 3-phase, 3-arm double–blind, placebo-controlled trial, conducted from February 2008 to January 2011 (**Figure 1** and **Figure S1**). Participants, recruited at two university medical centers (Montefiore Medical Center (Einstein), Bronx, NY, and Westchester Medical Center (New York Medical College), Valhalla, NY), gave informed written consent. The study was approved April 26, 2007 by The Institutional Review Board at Montefiore Medical Center (**Protocol S1**) and September 10, 2007 by The Committee of Human Subjects at New York Medical College (**Protocol S2**), and annually thereafter at both institutions. The protocol was also granted *full approval* by the Advisory Board of the General Clinical Research Center at Albert Einstein College of Medicine April 23, 2007. At the request of the Office of Research at New York Medical College, the protocol was also reviewed by the Division of Metabolism and Endocrinology, Center for Drug Evaluation and Research, of the FDA and received notice July 26, 2007 that it met “all requisites of CFR 32.2(b) (1) and accordingly” did not require an IND. Data and safety monitoring was conducted under the auspices of a non-study affiliate at Einstein. The study was registered at Clinicaltrial.gov, number NCT00618072 on February 5, 2008. CONSORT checklist is provided (**Checklist S1**). The authors confirm that all ongoing and related trials for this drug/intervention are registered.

### Subjects

Women meeting EMPOWIR study inclusion criteria (age 35–55, weight gain ≥20 pounds after the “twenties,” body mass index ≤35 Kg/m^2^) were invited to participate in a study to evaluate regimens to reduce insulin resistance. Key exclusion criteria were prior diagnosis of diabetes, use of an FDA approved or alternate medication for weight loss within the previous 6 months, known hepatic disease or alanine amino transferase (ALT) >40 U/L, known renal disease or creatinine >1.4 mg/dL, severe pulmonary disease, chronic acidosis of any etiology, congestive heart failure (>NYHA Category 1), history of known or suspected heart disease, untreated thyroid abnormality (TSH≤0.2 or ≥4 mIU/L), pseudotumor cerebri, contemplation of pregnancy, or other impairment that might preclude active study participation. Prospective study candidates completed a 400-item health history form. The survey included demographic variables, detailed family history, medical systems review, lifetime weight and dietary history, assessment of appetite, eating behaviors and exercise patterns and two widely validated psychometric instruments: the Center for Epidemiologic Studies for Depression (CES-D) and the CAGE (Cut-down, Annoyed, Guilty, and Eye opener) scale for alcohol use. Study eligibility was subsequently evaluated with a standard oral 75 gram glucose tolerance test (GTT) with insulin levels. Area-under-the-curve (AUC)-insulin was calculated by trapezoidal approximation [24]. As in earlier studies of midlife women [13], [25], [26], hyperinsulinemia was defined as an AUC-insulin ≥100 µU/ml, or a peak insulin response ≥60 µU/ml, along with normal fasting and 2-hour glucose responses. Subjects meeting study inclusion criteria also completed a brief weight loss readiness questionnaire to assess goal weight objectives, life circumstances, and social support structure.

### Randomization and Interventions

#### The Dietary Protocol

Prior to randomization, study subjects were required to participate in a 1 month lead-in phase consisting of four weekly 75 minute small group workshops conducted by a single study nutritionist (LK) at each of the study sites. Participants were advised that the goal of the workshops was the promotion of lifelong dietary and behavioral changes for long term weight maintenance based on implementation of the EMPOWIR dietary intervention. Workshops were scripted with a specific curriculum to ensure uniformity across sites. Each workshop featured a brief (15–20 minute) health education module in lecture format, followed by group discussion. The initial lecture defined insulin resistance and hyperinsulinemia, their relationship to diabetes and metabolic syndrome, and introduced the concepts underlying the carbohydrate modification of the EMPOWIR diet. Health education materials and detailed dietary information were distributed along with sample meal plans, menus, and recipes to optimize healthy food choices and facilitate implementation of the core prescribed dietary changes. Workshop participants also received instruction in the maintenance of eating records and exercise logs, appetite and fullness rating scales, and basic cognitive behavioral strategies based on the LEARN program [27], and used in the Women’s Health Initiative. Eating records were reviewed by the study nutritionist at each visit. Study coordinators attended selected workshops to enhance continuity of patient care and the study nutritionist provided email access to study participants to address questions and problems that arose between workshops. Study subjects were advised not to modify their exercise level or estrogen replacement for the duration of the study.

The EMPOWIR dietary intervention is a food exchange program with approximately 40–45% carbohydrates, 35–40% protein, and 15–20% fat, promoting increased intake of vegetables, low-glycemic index fruits, low-fat protein and dairy products, elimination of all added simple (free) sugars, and the notable restriction of three allowable additional servings of daily carbohydrates (starches) to after 4PM. The diet integrates important features of popular low-fat and carbohydrate-restricted diets and the Mediterranean Diet. Daily servings in each category are summarized with a simple 4-3-2-1-food pyramid and, with the exception of the vegetables, typically range between 80 and 120 calories, providing a total daily caloric intake of approximately 1300–1500 calories and a planned 600 kcal/day deficit (**Figure S2**). To simplify adoption of the diet and enhance compliance, study participants were instructed to carefully measure fats and estimate the portion size of carbohydrate and protein servings based on visual cues, but were not otherwise required to weigh and measure each item. They were encouraged to select from a wide variety of healthy vegetables, high fiber/low-glycemic index fruits, whole grains and legumes, fish, poultry, and low-fat dairy products, and to limit fats to monounsaturated fats.

Study subjects were advised that the stated study target goal was elimination of breakfast and lunchtime starches a minimum of 6 days out of 7 based on a review of written eating records, as available.

### Randomization and Study Protocol

Participants were randomly assigned to receive placebo, metformin, and metformin plus low dose rosiglitazone on a 1∶1∶1 ratio. Medication initiation was based on a complex dosage escalation program designed to deliver 500 mg of standard (i.e., non-extended release formula) metformin twice daily (or placebo) plus 2 mg of rosiglitazone (or placebo) for the first two weeks, with the addition of 500 mg metformin (or placebo) at week 3, and 500 mg metformin plus 2 mg rosiglitazone (or placebo) at week 4. Subjects who experienced intolerable gastrointestinal symptoms were instructed to contact their study coordinator for modification in the dosage escalation schedule. The randomization code was generated by the pharmacist responsible for clinical trials at Westchester Medical Center and was not broken until after the last patient completed the final 12-month follow-up visit.

Patients were seen at 1-, 2-, 3-, and 6-months following randomization (Phase 1) and at 1-, 2-, 3-, and 6- months following treatment reassignment (Phase 2: re-randomization of placebo group into one of the two active comparator arms) for a total of 8 follow-up visits (**Figure S1**), to record anthropometric measurements and hunger and appetite ratings, and monitor side effects and medication adherence (based on pill count). Body weight was measured on a beam balance scale. Height was measured on a stadiometer. Waist circumference was recorded at the narrowest diameter between the xiphoid process and the iliac crest; hip circumference was defined as the widest diameter below the umbilicus. All measurements were performed three times and averaged by a single study coordinator at each of the two sites.

### Outcome Measures

The primary outcome measure was change in fasting insulin at 6 months. Pre-specified secondary outcomes were 6-month change in body weight, HOMA-IR, glucose, waist circumference, blood pressure, total and HDL- cholesterol, triglycerides, leptin, and adiponectin.

### Assays and Measurements

Other than adiponectin and leptin, which were measured in single batches, analytes were measured at appointed visits in CLIA certified hospital-affiliated laboratories or the ICTR biomarker research core at Einstein. Glucose, HDL, total cholesterol, and triglycerides were measured by enzymatic immunoassay on an AU400 chemistry auto-analyzer (Beckman-Coulter Corporation, Nyon, Switzerland) with commercially available enzymatic reagents. Glucose was measured using the hexokinase method. Triglyceride and total cholesterol were measured by direct enzymatic measurements, while HDL was separately measured using two reagent homogenous systems with selective detergents to homogenize the lipoprotein of interest. Insulin was determined with a Siemens Immulite assay with respective intra- and inter-CV’s 5.7 and 5.9%, and no cross reactivity to pro-insulin. Total adiponectin was measured with a commercial ELISA kit (Millipore/Linco Research, St. Charles, MO) in the laboratory of Dr. Philipp Scherer. Leptin was measured at Quest Diagnostics Nichols Institute (San Juan Capistrano, CA) with a Millipore RIA. Components of the metabolic syndrome and relevant covariates were assessed using NCEP guidelines [28]. Homeostasis model assessment for insulin resistance (HOMA-IR) was calculated by the formula: fasting insulin (µU/ml) times fasting glucose (mM/L) divided by 22.5 [29].

### Sample Size Calculations and Statistical Analysis

Sample size calculations were provided by The Department of Epidemiology and Biostatistics, School of Public Health, NYMC. Using an analysis of covariance, a sample size of 60 patients (20 per group) was proposed to provide at least 80% power to detect a moderate to large effect size (*f* = .40) for a two-sided test of significance at a critical value of.05–assumptions and additional detail, page 12, **Protocol S1**, and pages 8–9, **Protocol S2**. The study was not powered for subgroup analysis by race or menopausal status.

Paired t-tests were used to compare within-group mean differences at baseline (prior to initiation of the study diet) and at 6 months following randomization for each of the 3 comparator groups. ANCOVA by baseline covariates were used to assess between group mean differences. Multivariate models were used for covariate adjustment by age, race, menopausal status, initial BMI group, and study site (SPSS 19.0). No imputation of missing values was used.

## Results

### Population characteristics and disposition

Four hundred and fifty five potential subjects were screened, with exclusion of 346, predominantly due to age and BMI (**Figure 1**). GTT’s were performed in 109 subjects with exclusion of a total of 41: 24 with normal glucose and insulin curves; 11 with glucose impairment (fasting blood sugar ≥100 mg/dL, 2 hour ≥140 mg/dL or both); 4 with overt diabetes; and 2 with incomplete data. All 68 subjects who met metabolic study criteria (AUC-insulin ≥100 µU/ml) attended and completed the lead-in phase nutrition workshops. However, 22 participants withdrew just prior to randomization, 19 due to reported concern over possible assignment to the rosiglitazone arm, following widespread media coverage of increased heart disease risk with rosiglitazone. The final study population consisted of 46 participants. After exclusion of two study completers due to clinical conditions which appeared *de* *novo* (asthma requiring high dose prednisone and growth hormone deficiency diagnosed mid-study), 44 subjects were included in the final dataset.

Mean age (SE), body weight, and BMI of the study participants were, respectively, 46.6±1.0 years, 82.4±1.6 kg, and 30.5±0.04 kg/m^2^ with comparable medians denoting essentially normal distribution of these variables (**Table 1** **, Figure S3**). Seventeen (38.6%) were black or Hispanic; 24 (57.1%) were college educated; 13 (29.5%) were health care professionals (**Table 2**). Twenty-six (59%) were peri- or postmenopausal and 27 (61.4%) had no family history of diabetes. Twenty-five (61.4%) exercised 3 or more times per week and only 4 were current smokers. Medication use included: anti-hypertensives in 10, thyroid supplements in 8, anti-depressants in 5, and estrogen replacement in two subjects. Mean baseline glucose, fasting insulin, HbA1C, and HOMA-IR were, respectively, 86.7±1.1 mg/dL, 10.7±0.7 µU/ml, 5.5±0.04%, and 2.3±0.2.

**Table 1:** Baseline metabolic characteristics by comparator groups.

	Placebo	Metformin	Metformin+Avandia	Total	
Parameters	Mean±SE	Mean±SE	Mean±SE	Mean±SE	Median
	(n = 16)	(n = 14)	(n = 14)	(n = 44)	
Age (yrs)	45.5±1.53	47.07±1.84	48.57±1.62	46.6±1.0	47.5
Weight (kg)	83.68±2.59	83.92±2.47	79.41±3.43	82.4±1.63	85.5
Body Mass Index (kg/m^2)^	30.15±0.70	31.26±0.71	29.95±0.91	30.5±0.04	30.4
Waist circumference (cm)	97.24±2.31	93.11±1.63	92.01±2.42	37.3±0.5	38.1
Systolic BP (mm Hg)	119.94±2.97	115±3.26	119±5.14	118.2±2.10	118
Diastolic BP (mm Hg)	76.87±2.08	73.07±2.28	74.54±3.44	75.4±1.4	78
Fasting glucose (mg/dL)	87.88±1.17	88.86±2.41	83.93±2.22	86.7±1.1	88
2-hour glucose (mg/dL)	107.14±5.65	106.57±4.82	101.43±7.03	106.3±3.30	108
Hemoglobin A1C (%)	5.55±0.07	5.39±0.08	5.52±0.08	5.5±0.04	5.5
Fasting insulin (µU/ml)	10.43±1.04	11.99±1.61	9.58±1.25	10.7±0.7	9.7
Peak insulin (µU/ml)	119.29±9.61	123.44±15.73	108.86±10.32	120.1±7.00	110
AUC insulin (µU/ml)	142.08±14.35	181.4±23.48	147.7±13.76	161.0±10.4	150.3
HOMA-IR	2.22±0.22	2.48±0.35	2.13±0.29	2.3±0.2	2
QUICKI	0.34±0.005	0.34±0.01	0.34±0.01	340.0±0.003	340
Total cholesterol (mg/dL)	174.69±5.49	201.5±9.74	204.09±11.58	191.1±5.6	182
HDL-cholesterol (mg/dL)	51.57±2.79	65.33±5.63	62.36±2.89	58.9±2.4	58.5
LDL-cholesterol (mg/dL)	102.14±5.03	114.08±5.99	125.91±10.14	111.9±4.3	110.5
Triglycerides (mg/dL)	92.92±8.75	110.92±24.42	78.73±10.67	95.0±9.3	82.5

**Table 2:** Sociodemographic characteristics by comparator groups.

Categorical variables			n (%)
	Placebo	Metformin	Metformin & Avandia
Family history of diabetes			
No	13(81.3)	7(50)	7(50)
Yes	3(18.8)	7(50)	7(50)
Educational Status**			
≥4 years of college	6(37.5)	9(64.3)	9(64.3)
<4 years of college	10(62.5)	4(28.6)	4(28.6)
Marital status			
Married or living with significant others	6(37.5)	10(71.4)	7(50.0)
Single (divorced, widowed or never married)	10(62.6)	4(28.6)	7(50.0)
Race			
white	8(50.0)	8(57.1)	8(57.1)
black	4(25.0)	3(21.4)	3(21.4)
Hispanic origin	3(18.8)	2(14.3)	2(14.3)
others	1(6.3)	1(7.1)	1(7.1)
Menopausal status***			
Premenopausal	5(31.3)	5(35.7)	5(35.7)
Perimenopausal	3(18.8)	2(14.3)	3(21.4)
Postmenopausal	7(43.8)	6(42.9)	5(35.7)
Exercise level**			
No exercise	8(50.0)	4(28.6)	5(35.7)
3 or more mild-moderate exercise/week	4(25.0)	7(50)	5(35.7)
3–4 sessions strenuous exercise/week	2(12.5)	2(14.3)	0(0)
5 or more exercise sessions/week	1(6.3)	1(7.1)	3(21.4)
Smoking			
Never smoked	10(62.5)	8(57.1)	7(50)
Former smoker	6(37.5)	3(21.4)	6(42.9)
Current smoker	0(0)	3(21.4)	1(7.1)
Anti-hypertensive medication*			
No	12(75.0)	11(78.6)	10(71.4)
Yes	3(18.8)	3(21.4)	4(28.6)
Anti-depressant medication*			
No	13(81.3)	14(100)	11(78.6)
Yes	2(12.5)	0(0)	3(21.4)
Thyroid supplements			
No	12(75.0)	13(92.9)	11(78.6)
Yes	4(25.0)	1(7.1)	3(21.4)
Estrogen replacement			
No	16(100)	13(92.9)	13(92.9)
Yes	0(0)	1(7.1)	1(7.1)
Health care professional			
No	11(68.8)	9(64.3)	11(78.6)
Yes	5(31.3)	5(35.7)	3(21.4)

*Responses declined or otherwise unrecorded in 1 participants.

**Responses declined or otherwise unrecorded in 2 participants.

***Responses declined or otherwise unrecorded in 3 participants.

### Follow-up data

Forty one of 46 of the subjects (89%) returned for the 6-month visit. However, the final dataset consisted of 44 subjects after exclusion of 2 study completers, as previously noted. Mean BW reduction was significant and comparable in D, M and MR groups (84.1 to 80.0 (4.7%), 85.1 to 80.4 (5.4%), and 81.8 to 77.5 (5.5%), *p’s* respectively. 049, .002, .032) (**Table 3** **, Figure S4**). Fasting glucose and insulin declined significantly only in the M group (89.9 to 84.0 mg/dL and 12.5 to 8.0 µU/ml, *p’s* = .034 and.026). HOMA–IR decreased in both M and MR groups (2.5 to 1.6 and 1.9 to 1.3, *p’s*.054 and .013). Other improved MS measures included increased HDL in the D and M groups (49.3 to 56.5 and 61.6 to 70.1 mg/dL, *p*’*s* = .016 and .030*)*; reductions in waist circumference in D and MR groups (97.5 to 93.1 and 93.1 to 87.5 cm, *p’s* = .052 and .005); systolic blood pressure in the M group (114.3 to 107.2 mm Hg, *p = *.001); and diastolic blood pressure in the D group (75.9 to 71.7 mm Hg, *p = *.025). LDL increased less in the M than the D or MR groups (111.2 to 111.3 mg/dL, *p* = .044, ANCOVA (with covariate adjustment by baseline values) with 21% of the variance explained by the study group. Total adiponectin increased from 11.1 to 18.5 µg/mL in the MR group *(p*<.001) and was not significantly changed in the D and M groups; baseline covariate adjusted ANCOVA *(p*<.001), with 56% of the variance explained by study group.

**Table 3:** Metabolic parameters at baseline and 6 month followup in 3 comparator groups.

	EMPOWIR diet and Placebo (D)				EMPOWIR diet and Metformin (MR)				EMPOWIR diet and Metformin plus Rosiglitazone (MR)			
	Baseline	6 months	Mean difference	*P* value*	Baseline	6 months	Mean difference	*P* value*	Baseline	6 months	Mean difference	*P* value*
**Parameters**												
Weight (kg)	84.1(2.8)	80.0(2.7)	–4.1	.049	85.1(2.4)	80.4(2.1)	–4.7	.002	81.8(3.2)	77.5(4.0)	–4.3	.032
Waist (cm)	97.5(2.3)	93.1(2.1)	–4.4	.052	92.9(1.8)	90.4(1.9)	–2.5	.143	93.1(2.1)	87.5(3.2)	–5.6	.005
SystolicBP (mm Hg)	117.5(2.9)	113.8(2.9)	–3.6	.265	114.3(3.4)	107.2(3.0)	–7.2	.001	118.7(6.0)	114.2(4.7)	–4.5	.389
DiastolicBP (mmHg)	75.9(2.6)	71.7(2.3)	–4.2	.025	75.3(2.0)	72.7(2.3)	–2.5	.162	76.9(4.2)	74.3(3.8)	–2.6	.562
Fastingglucose (mg/dl)	88.8(1.3)	84.9(2.2)	–3.8	.150	89.9(2.3)	84.0(1.8)	–5.9	.034	83.9(2.4)	80.8(1.4)	–3.1	.308
Fastinginsulin (µU/ml)	11.2(1.4)	8.1(1.3)	–3.1	.181	12.5(2.0)	8.0(1.2)	–4.5	.026	8.7(1.5)	6.3(0.9)	–2.4	.063
HbA1c (%)	5.4(0.1)	5.3(0.1)	–0.1	.309	5.4(0.1)	5.3(0.1)	0	.631	5.4(0.1)	5.4(0.1)	0	.645
HOMA-IR	2.3(0.2)	1.5(0.1)	–0.8	.142	2.5(0.4)	1.6(0.3)	–0.9	.054	1.9(0.3)	1.3(0.2)	–0.6	.013
Totalcholesterol(mg/dl)	176.0(5.3)	183.6(7.0)	7.5	.360	190.2(7.5)	202.0(7.0)	11.8	.206	200.9(12.0)	220.4(12.5)	19.5	.030
HDL (mg/dl)	49.3(2.3)	56.5(3.5)	7.2	.016	61.6(5.7)	70.1(5.8)	8.5	.030	60.1(3.2)	68.3(6.2)	8.2	.150
LDL (mg/dl)**	105.6(5.0)	108.1(5.6)	2.5	.602	111.2(7.9)	111.3(6.5)	0.1	.991	124.2(10.1)	130.2(8.8)	6.0	.373
Triglycerides(mg/dl)	90.1(9.5)	95.2(10.7)	5.2	.648	88.0(11.3)	103.1(13.0)	15.1	.094	82.6(11.2)	109.2(18.3)	26.6	.054
Adiponectin***	9.3(1.3)	10.6(1.2)	1.3	.092	10.6(1.4)	10.9(1.7)	0.3	.730	11.1(1.0)	18.5(1.5)	7.4	<.001

*p’s represent within group mean differences determined by paired t-tests.

**p = .044 for between group mean differences determined by ANCOVA by baseline covariate.

***p≤.001 for between group mean differences determined by ANCOVA by baseline covariate.

Mean leptin reported in all subjects with progressive weight loss and available leptin samples (n = 15) did not decline at 6 months; randomization: 26.15±2.01 ng/ml *vs.* 6 months: 25.99±2.61 ng/ml, *p* = .907 (**Table 4**).

**Table 4:** Body Weight, Leptin, and ratio Lp/BW in all 15 study subjects with progressive weight loss and available samples at Screening and 6 month visits.

Variable	Baseline^a^ (mean±SE)	6 month	Mean difference	P Value
Body weight (kg)	80.16±2.15	77.51±2.21	–2.65±1.00	.019
Leptin (ng/ml)	26.15±2.01	25.99±2.61	.17±1.40	.907
Leptin/body weight ratio (ng/ml/kg)	.3353±.03	.3327±.03	–.0059	.710

a Baseline (prior to nutritional intervention).

### Safety and compliance

No significant treatment emergent side effects were reported in the active treatment groups during the trial. The gradually escalating metformin dose was well tolerated; 5 subjects reported “mild” gastrointestinal (GI) problems; two reported “severe” GI symptoms – one of whom (with pre-existing ulcerative colitis) dropped out of the study. One patient with a prior history of hypertension was withdrawn due to elevated blood pressure recordings after randomization, but was later confirmed to have been in the placebo arm; an additional patient withdrew prior to initiation of study medication due to pregnancy.

## Discussion

This analysis demonstrates that (1) a significant percentage (68 of 109, 62.3%) of diverse, apparently healthy, overweight and mildly obese normoglycemic women with midlife weight gain have ascertainable hyperinsulinemia; (2) EMPOWIR’s easily implemented carbohydrate modified diet (CMD) produced significant weight loss–5% at 6 months – alone, and in combination with metformin and metformin plus low-dose rosiglitazone in this distinct study population; (3) Metformin enhanced the reduction of fasting insulin at 6 months in study subjects with diet induced weight loss.

The results provide support for *a priori* study hypotheses and our earlier reports suggesting that undetected insulin elevations are common among apparently healthy, non-diabetic women with midlife weight gain, and that early, intensive, targeted, interventions can reverse weight gain and reduce insulin levels. The treatment rationale and findings are consistent with an emerging consensus implicating insulin as a cause, as well as a consequence, of weight gain and the corollary that “lowering insulin secretion in hyperinsulinemic individuals may be beneficial” [17], proposed in major reviews [16]–[18], and increasingly documented in clinical [19], [20] and laboratory studies [21]–[23]. Reductions in both 6-month fasting insulin and HOMA-IR with metformin, and in HOMA-IR with combination therapy, suggest that insulin sensitizing medications may confer additional benefit beyond weight loss, in populations with documented insulin elevation and presumed diabetes risk.

To the best of our knowledge EMPOWIR is the first trial to specifically tailor and assess combined dietary and therapeutic options in subjects with documented insulin elevations. The weight reducing properties of metformin in non-diabetic subjects have been well delineated in prior studies [30]–[32]. However, the majority of these studies did not include a dietary intervention and the few metformin studies which included diets focused on caloric reduction [33]–[35] rather than carbohydrate modification. The EMPOWIR diet was explicitly designed to minimize glucose excursions, reduce postprandial insulin elevations, and modulate appetite, embodying findings from other clinical and laboratory investigations. These included: the demonstration of decreased *ad libitum* intake following high protein compared to high carbohydrate breakfasts [36]; data from clinical trials of low glycemic index diets [37], [38]; and studies of modulation of ghrelin and other key regulators of appetite and satiety with protein *vs*. carbohydrate intake [39].

The optimal diet for weight reduction remains an area of profound ongoing controversy. The EMPOWIR diet incorporates core elements of both low-fat and carbohydrate restricted diets within the basic framework of the Mediterranean diet, increasingly recommended for individuals with, or at risk for diabetes [40]. The diet differs from the traditional Mediterranean Diet in its inclusion of two daily low-fat dairy servings –reported to enhance weight loss in trials of dietary calcium in obese adults [41], and its reduction in total energy intake to approximately 1300–1500 Kcal/day.

The key features of the CMD are its novel macronutrient composition, 40–45% carbohydrates, 35–40% protein, and 15–20% fat – differing from other conventional diets (**Figure S5**); the notable restriction of three allowable additional servings of daily carbohydrates (starches) to after 4PM; and the inclusion of a 300–350 calorie weekly treat to diminish deprivation and boredom, improve dietary adherence, and encourage planning and behavioral change. Low glycemic load diets may be particularly important for weight loss in subjects with elevated insulin secretion, as concluded in an 18 month study of obese young adults [38] and advocated in a recent review on carbohydrate restriction for the “significant percentage of the population with insulin resistance or … metabolic syndrome” [42].

Dietary comparison studies have demonstrated the efficacy, safety, and metabolic advantages of high protein diets. Reported benefits in *ad libitum* trials have included reduction in fat mass, triglycerides, and free fatty acids in healthy overweight and obese subjects [43]; increased weight loss in severely obese adolescents [44]; reduction in body weight and diastolic blood pressure in overweight and obese adults [45]; and enhanced insulin sensitivity and reduced inflammatory adipokines in obese non-diabetic females [46]; along with increased energy expenditure and lean mass in a rigorously controlled overconsumption study in young adults [47]. These effects have been attributed to the ability of protein “to suppress hunger and induce satiety,” highlighted in recent commentaries [48] [49]. Consistent with these observations, our study subjects uniformly reported a decrease in hunger and food cravings when they postponed consumption of the three allowable starches until 4PM, replicating reports of enhanced satiety with carbohydrates eaten predominantly at dinner, in a recent randomized trial of obese Israeli police officers [50].

The EMPOWIR dietary intervention was easily implemented– requiring participation in a total of only 4 group nutritional workshops, considerably fewer than in most other lifestyle modification studies. The ease of adoption may have been due to the simplicity of the diet or to the designated core objectives and content of the educational materials – its specific focus on reduction of high glycemic index foods in a cohort with documented glucose-mediated hyperinsulinemia. This presumably also contributed to the study’s high retention rate–41 of 46 study participants (89%) returned for the 6-month visit.

Additional study findings include a robust increase in adiponectin in the MR group – consistent with the mode of action of rosiglitazone – and the noteworthy preservation of leptin levels in a subset of study subjects with progressive weight loss at all study intervals, as reported previously [51]. Maintenance of leptin despite weight loss was thoroughly unexpected, in view of the well documented central role of leptin decline as a primary mediator of weight regain in body weight regulation [52], [53]. The observed differences may relate to the nature of the dietary intervention, the use of metformin, or to intrinsic characteristics of our study cohort. Modulation of leptin sensitivity either by metformin, as previously demonstrated [54], or secondary to reduction in insulin levels, as proposed by other investigators [20], may also have contributed to these results. Clearly, additional research is required to confirm these findings and elucidate potential underlying mechanisms.

Strengths of the study include (1) the ethnic diversity of the study cohort, supporting potential generalizability to a broad range of midlife women with demonstrable risk for diabetes and related comorbidities; (2) the low rate of medication- related treatment emergent events, suggesting that, as in the CANOE Trial and Xian Study [55], [56], low dose rosiglitazone may provide benefit in selected high risk populations; and (3) the 89% 6-month study retention rate.

The major study limitation was its high (33%), unanticipated, dropout rate immediately prior to randomization, which compromised study power, precluding use of the more robust ANCOVA for analysis of the primary study outcome variable. Notably, all 68 subjects who met study eligibility criteria based on glucose tolerance testing, completed the mandatory lead-in nutritional phase – exceeding sample size estimates, 20 per comparator group for a total of 60 subjects at randomization (**Figure S1**, **Protocol S1**, **Protocol S2**) whereas only 46 of the 68 were ultimately randomized to medication. Nineteen of the 22 subjects who withdrew at this study interval reported concern over possible assignment to rosiglitazone as the reason for withdrawal, in view of widespread media coverage of cardiovascular risks of the medication [57].

Women with progressive midlife weight gain are prime targets for early and aggressive preventive interventions, based on the association of weight gain with multiple deleterious life altering health outcomes [2]–[6]. Recent CDC data indicate a significant increase in the rate of obesity (from 31.5 to 38.1%, *p* = .006) among women aged 60 and older [58], which underscores the need to address increasing adiposity in this earlier, critical stage of the life cycle. The low cost and established long-term safety profile of metformin, and the efficacy and relative ease of adoption and durability of the carbohydrate modified diet, suggest that EMPOWIR’s dual regimen might provide new treatment models for midlife women with defined hyperinsulinemia.

In conclusion, the EMPOWIR diet independently, and in combination with insulin sensitizers, reduced body weight in diverse, overweight and obese, normoglycemic, hyperinsulinemic midlife women. However, superior reductions in 6-month fasting insulin in the metformin arm, and in HOMA-IR in both M and MR arms, suggest potential additive benefit with the addition of insulin sensitizing medications in populations with documented insulin elevation and presumed risk for diabetes and metabolic syndrome. Notably, leptin did not decline in a subset of 15 study subjects with progressive weight loss at the major study interval. Effective, easily implemented dietary interventions, alone and in combination with pharmacotherapies that target hyperinsulinemia in midlife women, merit additional investigation in larger scale long term studies.

## Supporting Information

Figure S1 **Overview of EMPOWIR Study Flowchart.** Summary of Screening, Lead-in, and Study phases for the EMPOWIR trial.
(TIF)

Figure S2 **Overview of the EMPOWIR Dietary Intervention.** Summary of 18 daily and 1 weekly servings of the EMPOWIR dietary intervention.
(TIF)

Figure S3 **Shapiro-Wilk’s Tests of Normality for Primary and Secondary Outcome Variables and Relevant Covariates.**
(PDF)

Figure S4 **Mean 6-month percentage changes key metabolic parameters by Comparator Group.** *p-*values reflect within group mean differences determined by paired t-tests * = ≤.05, * = ≤.01, *** = ≤.001 A. 6 month percentage change in body weight B. 6 month percentage change in fasting insulin C. 6 month percentage change in HOMA-IR D. 6 month percentage change in Waist Circumference E. 6 month percentage change in HDL cholesterol F. 6 month percentage change in Adiponectin.
(TIF)

Figure S5 **Comparison of Macronutrient Composition of EMPOWIR with Other Popular Diets.**
(TIF)

Checklist S1 **CONSORT checklist.**
(PDF)

Protocol S1 **Trial protocol as submitted to Albert Einstein College of Medicine IRB.**
(PDF)

Protocol S2 **Trial protocol as submitted to New York Medical College IRB.**
(PDF)
