# Supplementary figures and images for: Carbohydrate Modified Diet & Insulin Sensitizers Reduce Body Weight & Modulate Metabolic Syndrome Measures in EMPOWIR (Enhance the Metabolic Profile of Women with Insulin Resistance): A Randomized Trial of Normoglycemic Women with Midlife Weight Gain
Source: PLoS One. 2014 Sep 26;9(9):e108264. doi: 10.1371/journal.pone.0108264 (PMC4178125; doi:10.1371/journal.pone.0108264)

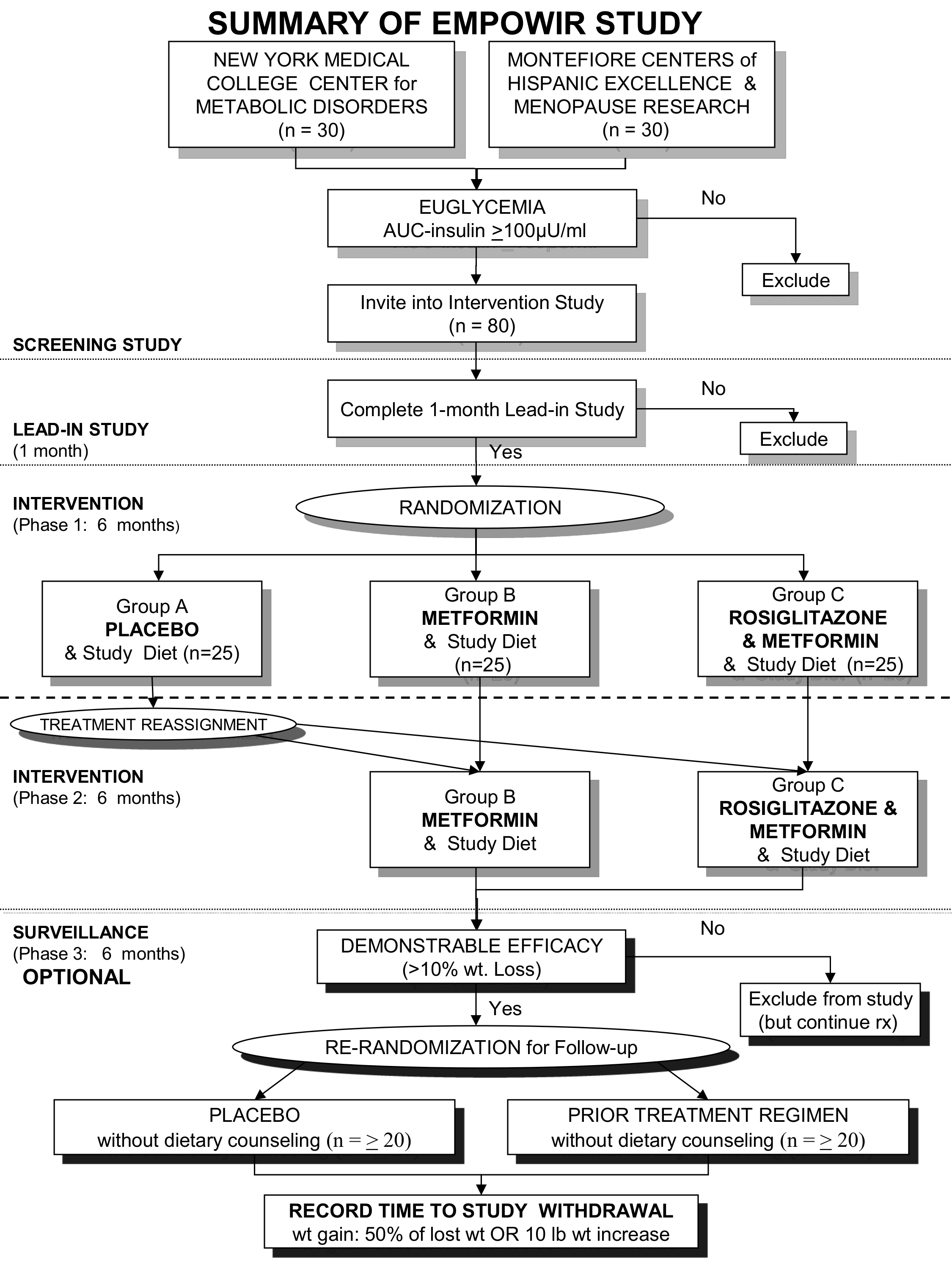

Supplement: Figure S1 — Overview of EMPOWIR Study Flowchart. Summary of Screening, Lead-in, and Study phases for the EMPOWIR trial. (TIF) [file pone.0108264.s001.tif]

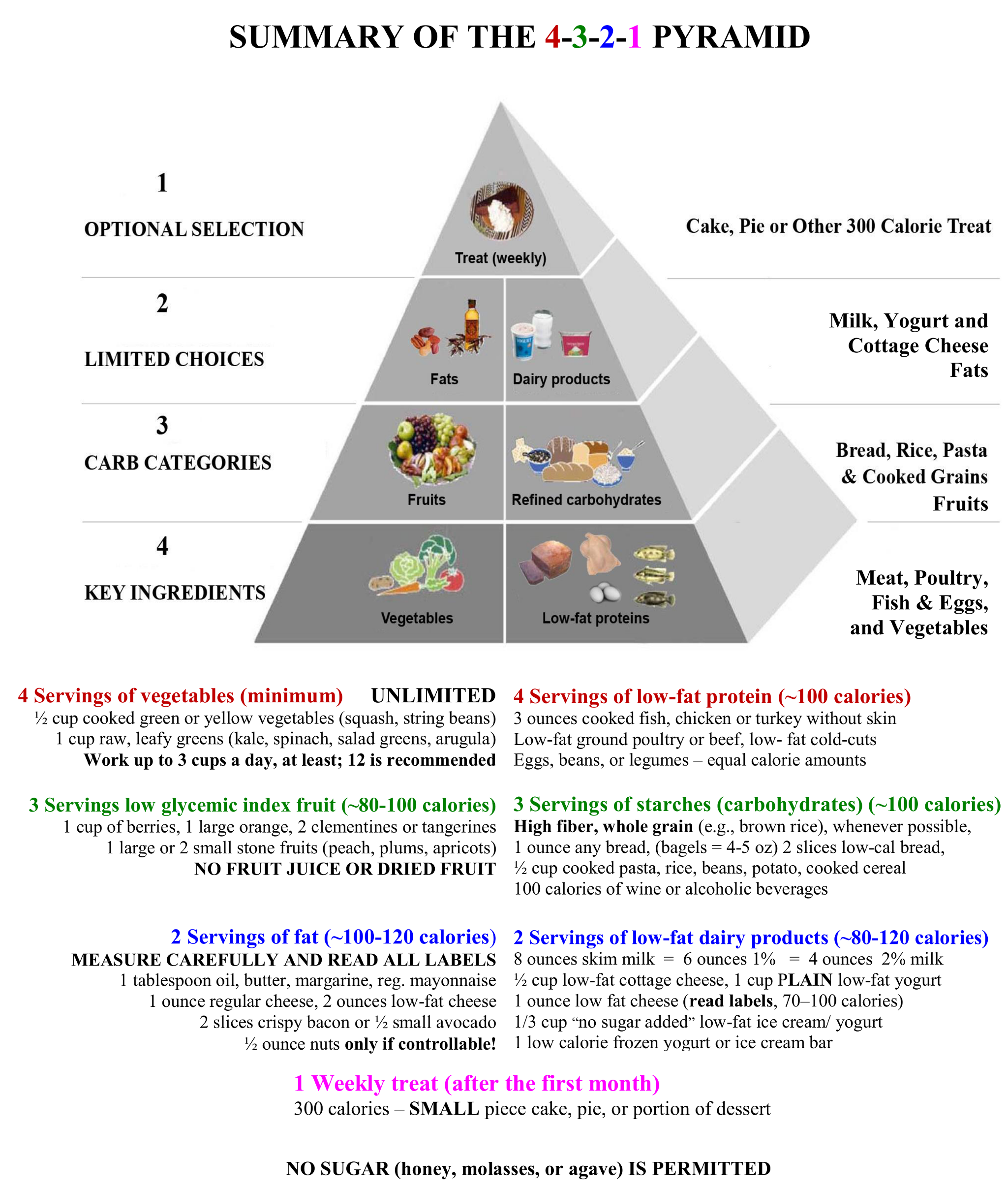

Supplement: Figure S2 — Overview of the EMPOWIR Dietary Intervention. Summary of 18 daily and 1 weekly servings of the EMPOWIR dietary intervention. (TIF) [file pone.0108264.s002.tif]

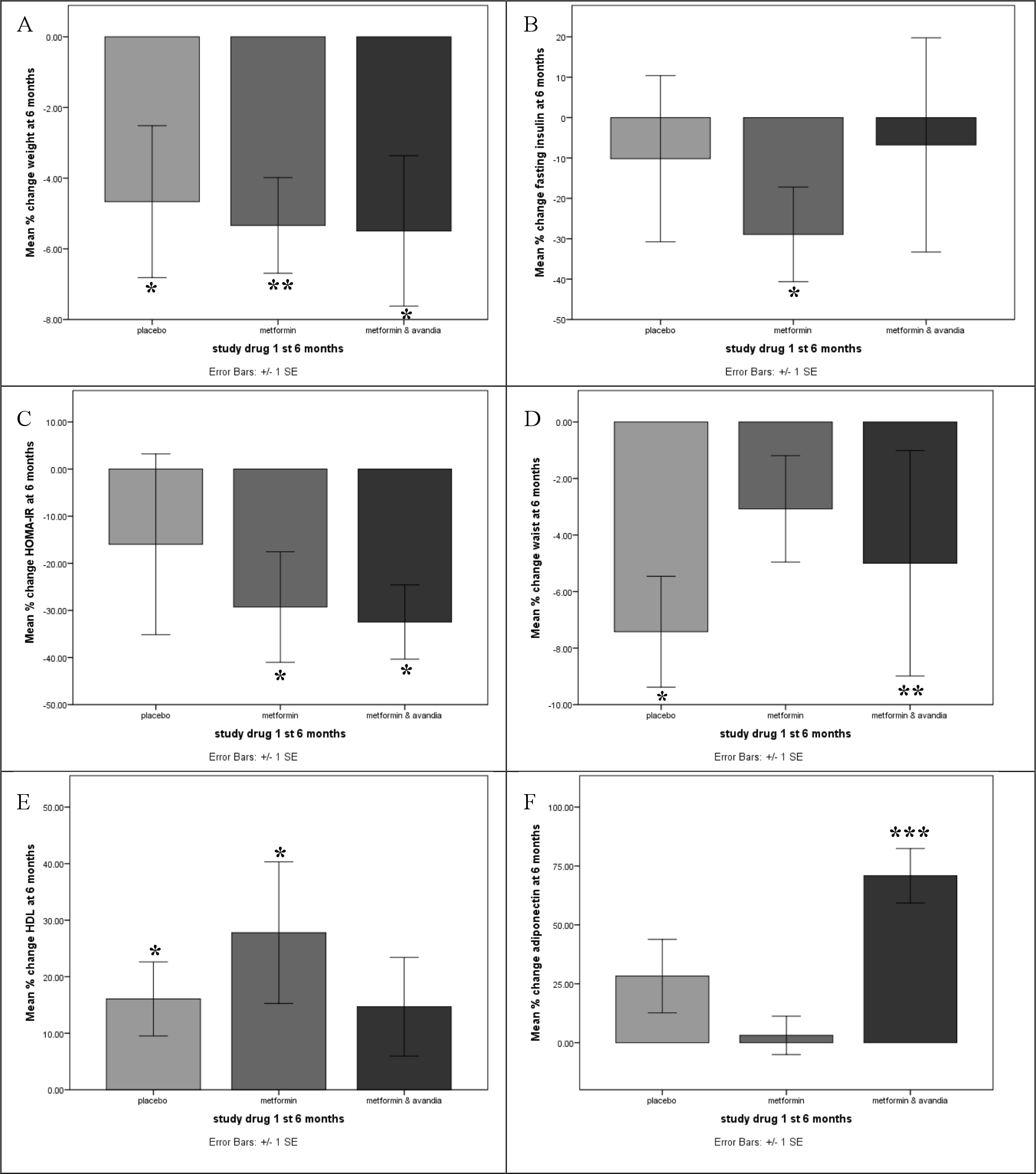

Supplement: Figure S4 — Mean 6-month percentage changes key metabolic parameters by Comparator Group. p-values reflect within group mean differences determined by paired t-tests * = ≤.05, * = ≤.01, *** = ≤.001 A. 6 month percentage change in body weight B. 6 month percentage change in fasting insulin C. 6 month percentage change in HOMA-IR D. 6 month percentage change in Waist Circumference E. 6 month percentage change in HDL cholesterol F. 6 month percentage change in Adiponectin. (TIF) [file pone.0108264.s004.tif]

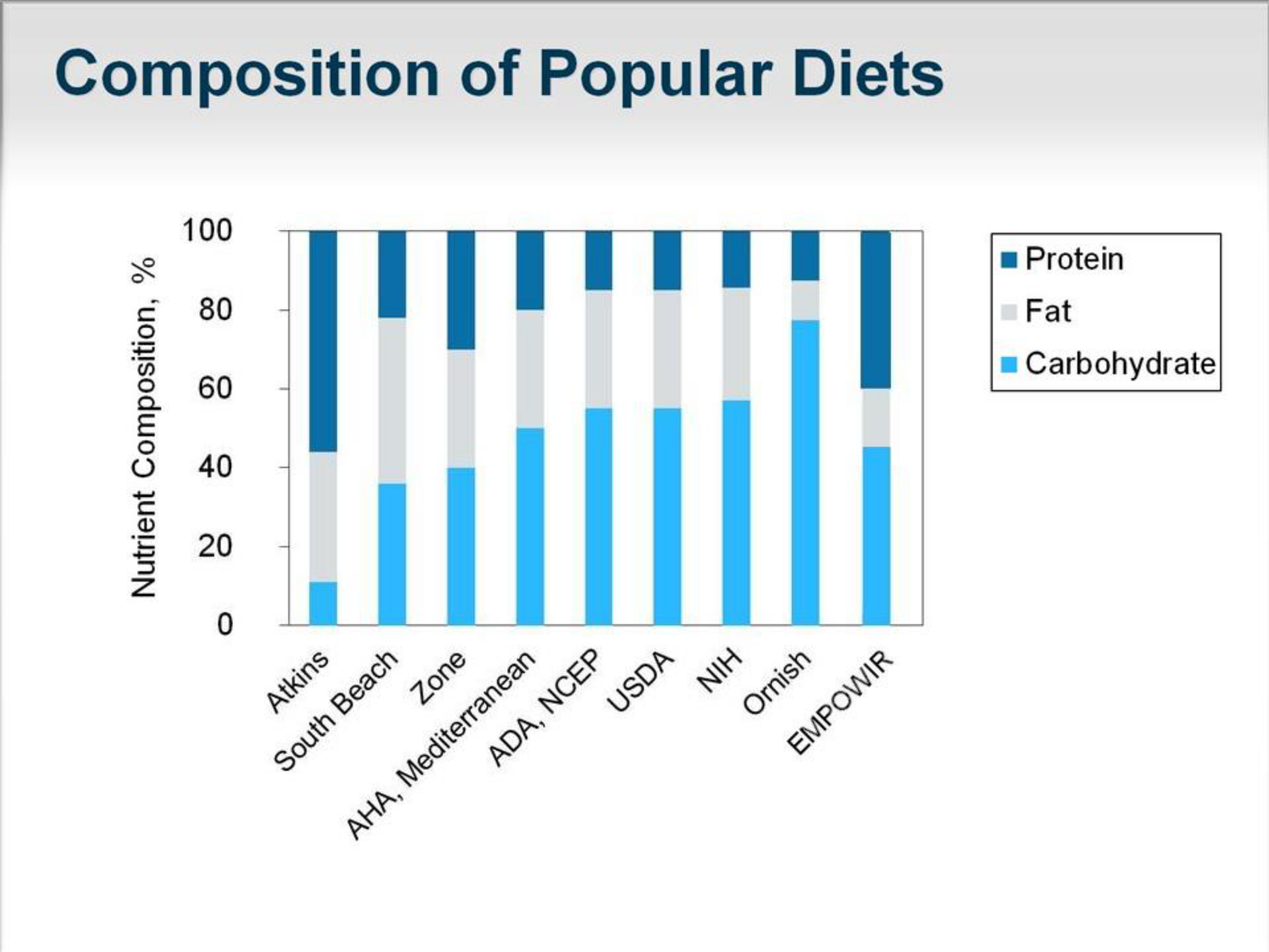

Supplement: Figure S5 — Comparison of Macronutrient Composition of EMPOWIR with Other Popular Diets. (TIF) [file pone.0108264.s005.tif]
