# Supplementary material for: Carbohydrate Modified Diet & Insulin Sensitizers Reduce Body Weight & Modulate Metabolic Syndrome Measures in EMPOWIR (Enhance the Metabolic Profile of Women with Insulin Resistance): A Randomized Trial of Normoglycemic Women with Midlife Weight Gain
Source: PLoS One. 2014 Sep 26;9(9):e108264. doi: 10.1371/journal.pone.0108264 (PMC4178125; doi:10.1371/journal.pone.0108264)
Supplement: Figure S3 — Shapiro-Wilk’s Tests of Normality for Primary and Secondary Outcome Variables and Relevant Covariates. (PDF) [file pone.0108264.s003.pdf]

Figure S6: Shapiro-Wilk's Tests of Normality for Primary and Secondary Outcome Variables and Relevant Covariates

Tests of Normality

study drug 1 st 6 months		Kolmogorov-Smirnov[a]			Shapiro-Wilk		
		Statistic	df	Sig.	Statistic	df	Sig.
screening/baseline weight in kg CORRECTED	placebo	.181	8	.200*	.947	8	.677
	metformin	.192	4		.975	4	.875
	metformin & avandia	.312	3		.896	3	.372
6 month weight in kg CORRECTED	placebo	.201	8	.200*	.958	8	.788
	metformin	.374	4		.808	4	.117
	metformin & avandia	.187	3		.998	3	.914
waist at baseline in cm	placebo	.251	8	.147	.829	8	.058
	metformin	.228	4		.926	4	.572
	metformin & avandia	.280	3		.938	3	.520
waist at 6 months in cm	placebo	.172	8	.200*	.965	8	.852
	metformin	.220	4		.940	4	.654
	metformin & avandia	.247	3		.969	3	.664
baseline SBP	placebo	.161	8	.200*	.934	8	.551
	metformin	.332	4		.853	4	.235
	metformin & avandia	.374	3		.778	3	.062
6 month SBP	placebo	.214	8	.200*	.904	8	.311
	metformin	.290	4		.863	4	.271
	metformin & avandia	.219	3		.987	3	.780
baseline DBP	placebo	.289	8	.047	.736	8	.006
	metformin	.331	4		.877	4	.325
	metformin & avandia	.341	3		.846	3	.230
6month DBP	placebo	.184	8	.200*	.889	8	.230
	metformin	.232	4		.968	4	.827
	metformin & avandia	.175	3		1.000	3	1.000
FBS converted to mmol/L (corrected)	placebo	.126	8	.200*	.965	8	.860
	metformin	.303	4		.791	4	.086
	metformin & avandia	.253	3		.964	3	.637

6-month fasting blood sugar mmol/L(corrected)	placebo	.192	8	.200*	.932	8	.538
	metformin	.295	4		.857	4	.250
	metformin & avandia	.196	3		.996	3	.878
Fasting insulin at baseline (avg)	placebo	.147	8	.200*	.982	8	.974
	metformin	.292	4		.928	4	.580
	metformin & avandia	.359	3		.811	3	.142
6mon fasting insulin	placebo	.178	8	.200*	.939	8	.605
	metformin	.198	4		.969	4	.836
	metformin & avandia	.346	3		.837	3	.206
Hb A-1-C	placebo	.269	8	.092	.815	8	.041
	metformin	.329	4		.895	4	.406
	metformin & avandia	.292	3		.923	3	.463
Hb A-1-C at 6 month	placebo	.148	8	.200*	.955	8	.761
	metformin	.262	4		.895	4	.408
	metformin & avandia	.292	3		.923	3	.463
HOMA-IR baseline(corrected and checked)	placebo	.185	8	.200*	.973	8	.921
	metformin	.207	4		.978	4	.891
	metformin & avandia	.356	3		.816	3	.154
HOMA-IR 6months (corrected)	placebo	.176	8	.200*	.969	8	.891
	metformin	.164	4		.987	4	.943
	metformin & avandia	.337	3		.855	3	.253
baseline CHOLB	placebo	.229	8	.200*	.887	8	.222
	metformin	.254	4		.921	4	.544
	metformin & avandia	.362	3		.804	3	.125
Cholesterol at 6 month	placebo	.142	8	.200*	.952	8	.732
	metformin	.170	4		.981	4	.909
	metformin & avandia	.214	3		.989	3	.801
baseline HDLB	placebo	.207	8	.200*	.874	8	.166
	metformin	.250	4		.950	4	.715
	metformin & avandia	.307	3		.904	3	.398

HDL at 6 month visit	placebo	.158	8	.200*	.932	8	.537
	metformin	.135	4		1.000	4	.999
	metformin & avandia	.228	3		.982	3	.743
baseline LDL	placebo	.196	8	.200*	.893	8	.250
	metformin	.276	4		.945	4	.684
	metformin & avandia	.370	3		.786	3	.082
LDL 6 month visit	placebo	.200	8	.200*	.906	8	.325
	metformin	.223	4		.945	4	.686
	metformin & avandia	.349	3		.832	3	.194
Triglycerides (mmol/L) bsln	placebo	.159	8	.200*	.969	8	.892
	metformin	.224	4		.949	4	.709
	metformin & avandia	.257	3		.961	3	.621
6month TG	placebo	.210	8	.200*	.910	8	.356
	metformin	.261	4		.892	4	.394
	metformin & avandia	.214	3		.989	3	.803
Adiponectin at baseline	placebo	.148	8	.200*	.986	8	.988
	metformin	.260	4		.952	4	.729
	metformin & avandia	.292	3		.923	3	.463
Adiponectin at 6 months	placebo	.202	8	.200*	.941	8	.623
	metformin	.321	4		.875	4	.317
	metformin & avandia	.235	3		.978	3	.714

*. This is a lower bound of the true significance.

a. Lilliefors Significance Correction
