# Supplementary material for: Carbohydrate Modified Diet & Insulin Sensitizers Reduce Body Weight & Modulate Metabolic Syndrome Measures in EMPOWIR (Enhance the Metabolic Profile of Women with Insulin Resistance): A Randomized Trial of Normoglycemic Women with Midlife Weight Gain
Source: PLoS One. 2014 Sep 26;9(9):e108264. doi: 10.1371/journal.pone.0108264 (PMC4178125; doi:10.1371/journal.pone.0108264)
Supplement: Protocol S1 — Trial protocol as submitted to Albert Einstein College of Medicine IRB. (PDF) [file pone.0108264.s007.pdf]

EMPOWIR (Enhance the **M**etabolic **P**rofile of **W**omen with **I**nsulin **R**esistance)

An unsolicited, investigator-initiated study – funded by GSK

STUDY INVESTIGATORS and PARTICIPATING INSTITUTIONS

Principal Investigator: Harriette R. Mogul MD MPH,
Associate Professor, Director of Endocrine Research, Department of Medicine
New York Medical College

Co-Principal Investigator: Ruth Freeman MD,
Professor of Medicine, Director Menopause Research and Treatment Center
Montefiore Medical Center, Albert Einstein College of Medicine

Co-Investigators:
Nerida Correida MD, *Associate Professor of Obstetrics & Gynecology*
Co-Director, Hispanic Center of Excellence
Albert Einstein College of Medicine

Beverly Williams Cleaves MD, *Associate Professor of Medicine*
Director, Diabetes Center of Excellence
University of Tennessee at Memphis

INTRODUCTION and STUDY RATIONALE

Midlife weight gain in women

Weight gain in midlife women has profound health implications. As first demonstrated by the Nurses Health Study, weight gain of 10 kg (22 pounds) or more after the age of 18 is associated with the development of Type 2 diabetes (1) and, after adjustment for relevant covariates, such as smoking, has a linear effect on age-adjusted cardiovascular,(2) (3) and all-cause mortality rates in women.(4) Moreover, excess adiposity negatively impacts job attainment, pay-scale and marital status(5) with a greater economic and social burden on women. Thus, midlife weight gain, long regarded an inevitable consequence of aging, is a harbinger of diabetes, and multiple additional adverse health and psychosocial outcomes. This suggests that midlife women with weight gain may be an important target for preventive interventions that address weight regulation and diminish risk for subsequent development of diabetes and cardiovascular disease.

Progressive weight gain that starts in the fourth and fifth decades is commonly reported by women from all ethnic and socio-economic groups.(6) Our previous data suggest that, in large and diverse subpopulations of healthy-appearing women, midlife weight gain may represent an early clinical manifestation of insulin resistance – demarcated by increased insulin response curves despite completely normal glucose tolerance tests.(7) We termed this disorder **Syndrome W**(8) to highlight its defining triad of **w**eight gain, **w**aist gain and **w**hite-coat hypertension in **w**omen along with its prominence as an alphabetic and chronologic antecedent to the far more familiar Syndrome X (now increasingly termed The Metabolic Syndrome). Presumably, as in other disorders of insulin action in younger women, including Polycystic Ovarian Syndrome (PCOS), early adrenarche, (9) and precocious puberty, Syndrome W represents a precursor of both diabetes and The Metabolic Syndrome at an early and optimal period for intervention.

Metformin – rationale for obesity management and metabolic risk reduction

Preliminary data from our first pilot study suggested that metformin, in combination with a hypocaloric, low-fat, carbohydrate modified dietary program produced significant and sustainable weight loss in women with Syndrome W, with notable reductions in fasting insulin levels (see *Preliminary Findings*)(10) Additional two to four year follow-up in an intention-to-treat analysis of consecutive women who lost ≥10% of their body weight after one year of the treatment regimen further suggests that this composite intervention prevents weight regain (11) that should further limit the onset of overt glucose impairment.

Our dual dietary medical protocol evolved from evaluation and treatment of several hundred patients seen in The Endocrine Faculty Practice, and The Obesity Research and Treatment Center at Westchester Medical Center over a ten year period and has been highly successful in a broad ethnic range of normoglycemic, hyperinsulinemic subjects. These include midlife women with weight gain; younger overweight-obese women with Polycystic Ovarian Syndrome; overweight men with upper body obesity and obese adolescents – populations, which have not been comparably treated in prior studies. The magnitude and duration of the treatment effect and its potential impact in the context of the current obesity and diabetes epidemics suggest that additional, more rigorous investigation should be undertaken. A randomized clinical trial of women with progressive and intractable weight spiral in the menopause transition could also evaluate hypothesis of other researchers implicating hyperinsulinemia as antecedent, as well as a consequence, of weight gain.(12-15)

The use of metformin in the primary treatment of obesity is consistent with findings from a wide variety of studies. These include clinical trials of insulin resistant subjects,(16) prospective studies of diabetics,(17) non-diabetic Europeans with central fat distribution,(18) insulin-resistant women with Polycystic Ovarian Syndrome,(19-22) men with Human Immunodeficiency Virus (HIV)-related lipodystrophy,(23) and children.(24) Metformin reduced total caloric intake in short-term, placebo-controlled experimental studies in both diabetics and non-diabetics (25;26) and metformin has many desirable, well delineated pharmacological actions. These include decreased hepatic glucose output,(27) and reduction of free fatty acids (FFA's)(28) – an important established link with central obesity(29) (30) and a critical component in the pathogenesis of insulin resistance via the augmentation of hyperinsulinemia in both the basal and post-prandial state.(31;32) The long-term safety profile of metformin and its association with cardiovascular risk reduction in numerous prospective studies(18;33-35) further support its unique potential as a therapeutic agent to treat obesity and diminish risk of Type 2 diabetes in midlife women with documented hyperinsulinemia.

The proposed study addresses a unique population of subjects at risk for diabetes and Metabolic Syndrome. Midlife women with weight gain, hyperinsulinemia and normoglycemia, documented by standard glucose tolerance testing, have not been the focus of prior treatment trials of either metformin or other insulin sensitizing medications. The Diabetes Prevention Program intervened at later stages of the progression to diabetes and did not include an arm for the combination of metformin with specific dietary strategies to modulate insulin overproduction. The DREAM Study which included lifestyle and hypocaloric diet also targeted subjects with impairments in glucose homeostasis.

The thiazolidinediones – additional therapeutic options to EMPOWIR (enhance the **metabolic profile of women** with **insulin resistance)**
PPAR agonists including thiazolidinediones (TZD's) are a relatively newer category of insulin sensitizers with increasingly wide and well-studied positive attributes, including improvements in lipid and blood pressure parameters, (36;37) redistribution of fat depots,(38) increased adiponectin secretion, and reduction of inflammatory and proinflammatory markers.(39) Clinical use of TZD's and metformin has been expanded to encompass additional disorders of insulin resistance, including PCOS, (40-42) (43) non-alcoholic hepatitis (NASH), and HAART (highly active antiretroviral treatment) – induced HIV lipodystrophy (44-46).

The combination of metformin and rosiglitazone (Avandamet®) is FDA-approved for the treatment of hyperglycemia in patients with Type 2 diabetes. Previous clinical research and recent laboratory data – metformin mediated reduction of hepatic LKB1in murine knockout models (Science, Nov, 2005) suggest that the two categories of insulin sensitizers have independent(47) and additive (38;48) mechanisms of action that could target and, ultimately, modulate the underlying pathogenesis of insulin resistance.

Comparison studies suggest that TZD's may have a greater insulin sensitizing action and provide greater reduction in hyperinsulinemia than metformin. However, due to increased adipocyte expression (and possible other mechanisms), weight gain is a common and undesirable side effect of TZD treatment. The addition of metformin to rosiglitazone, along with dietary strategies that reduce endogenous insulin production could prove an ideal therapeutic option to attenuate insulin resistance and preserve ß-cell function in high risk individuals. Early initiation of this dual regimen in normoglycemic subjects with documented hyperinsulinemia could have important implications for Syndrome W women and for an additional 25% of the adult US population estimated to have other early manifestations of The Metabolic Syndrome.

EMPOWIR differs in several respects from previously published and ongoing clinical trials of TZD's, metformin, or lifestyle intervention. The primary study question addressed is whether dual treatment regimens which modulate insulin action can reduce hyperinsulinemia and insulin resistance in high risk, but healthy-appearing normoglycemic, hyperinsulinemic subjects identified because of progressive, intractable, midlife weight gain. In contrast to other trials of thiazolidinediones, such as DREAM (49), ACCORD, BARI-2D, and PIPOD, which enroll patients with overt glycemic abnormalities, EMPOWIR specifically targets normoglycemic women from minority populations and assesses insulin rather than glucose parameters as the primary outcome measures.

Summary of Study Hypotheses

These related hypotheses are derived from clinical observations and emerging research on the role of insulin in the pathogenesis of obesity and related adverse health outcomes.

H_o: Compensatory hyperinsulinemia (independently or in association with abnormalities in insulin action, free fatty acid metabolism or other metabolic correlates) represents a common, generally undetected metabolic abnormality that promotes and sustains obesity in distinct subpopulations.

H_o: Even in the absence of overt glycemic disturbance, insulin elevations should be a primary treatment target of insulin sensitizing agents (along with other synergistic therapies that attenuate insulin resistance and reduce compensatory insulin secretion) in patients at risk for Type 2 diabetes and The Metabolic Syndrome.

H_o: Early identification of hyperinsulinemia and early initiation of insulin sensitizing agents could alter pathogenesis and disease progression in normoglycemic women (Syndrome W) and provide an important treatment model for additional populations at risk for Type 2 diabetes and Metabolic Syndrome

PRELIMINARY FINDINGS

1-year open label study (with 6-month follow-up surveillance) of the treatment protocol in 26 women (10)

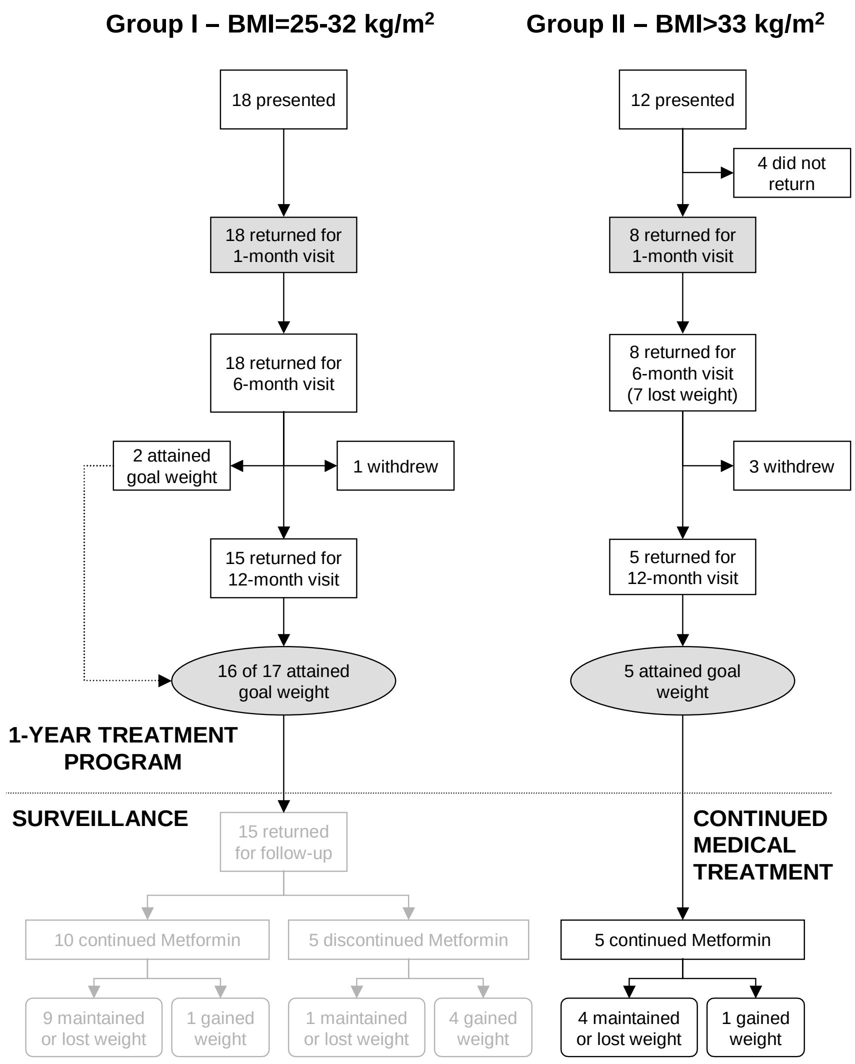

Figure 1: **Case series profile and summary of results**

Background: We conducted a retrospective analysis of a new obesity treatment protocol, metformin and hypocaloric, carbohydrate-modified diet, in a case series of high-risk, non-diabetic hyperinsulinemic women with progressive midlife weight gain (refractory to diet and exercise). **Methods:** 30 consecutive non-diabetic women with glucose-mediated area-under-the-curve (AUC-) insulin elevations (≥100μU/mL) in two Body Mass Index (BMI) categories (Group I: 25-32.9kg/m^2 and Group II: 33-41.7kg/m^2), presenting to an academic medical center-based weight reduction program, participated in a 1-year treatment program of metformin (mean daily doses of 1500mg/day (Group I) and 2000mg/day (Group II)) and carbohydrate-modified dietary regimens. Follow-up body weight (at 3-, 6-, and 12-months), percentage of patients meeting goal weight attainment (10% reduction in body weight or BMI normalization), and fasting insulin levels (as available) are reported in 26 women (18/18 in Group I and 8/12 in Group II) who returned for ≥1 follow-up visit.

Results: Significant weight loss was observed at 3, 6, and 12 months in both Group I (3.47 [SE 0.68], 6.41 [0.72], and 8.06 [0.96] kg, p's<0.0001) and Group II (4.4 [0.8], 9.7 [2.3], 15.1 [3.3], p=0.001, 0.004, 0.011). Twenty-five of 26 (96%) patients lost ≥5% of their body weight (BW) at 6-months and 21/26 (81%) patients lost ≥10% BW at 12-months. Post-treatment fasting insulin decrement (-35.5 [8.2]%) was the most significant predictor of 1-year weight loss. (R^2=0.656, regression coefficient=0.810, p=0.005). Surveillance data, available in 15/17 Group I patients who attained goal weight, demonstrated weight maintenance (within 1 kg) in the absence of direct medical supervision in 9 of the 10 patients who continued metformin, in contrast to weight gain (≥4kg or 50% of lost weight) in 4 of the 5 patients who discontinued metformin.

Conclusion: Progressive weight loss (96% of patients with 5% 6-month BW reduction; 81% with 10% 1-year BW reduction) was observed in 2 groups of high-risk, non-diabetic, hyperinsulinemic women, treated with metformin and carbohydrate-modified hypo-caloric diet; weight stabilization was seen in 9/10 patients who continued medication after the 1-year program.

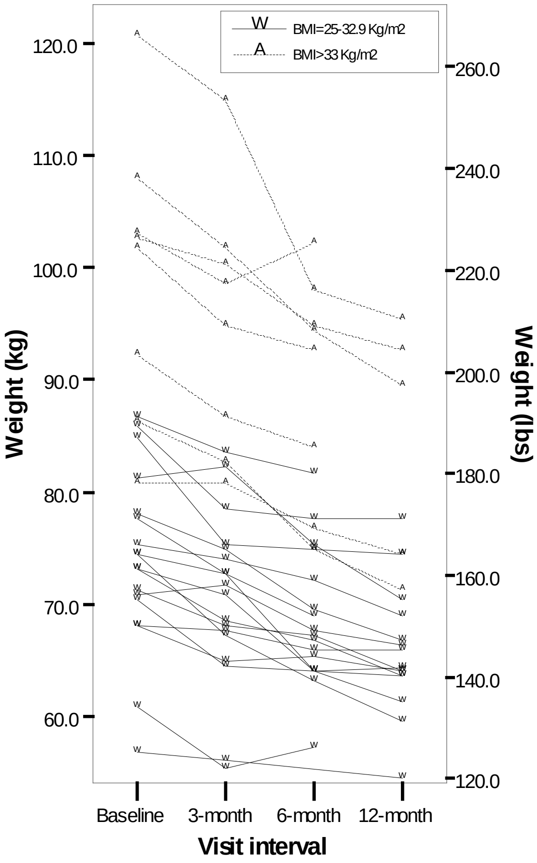

Figure 2. Body weight at specified treatment intervals following initiation of Metformin and dietary intervention in 26 patients. BMI = body mass index. *(Note: overlapping values account for decreased number of datapoints at 12 months.)*

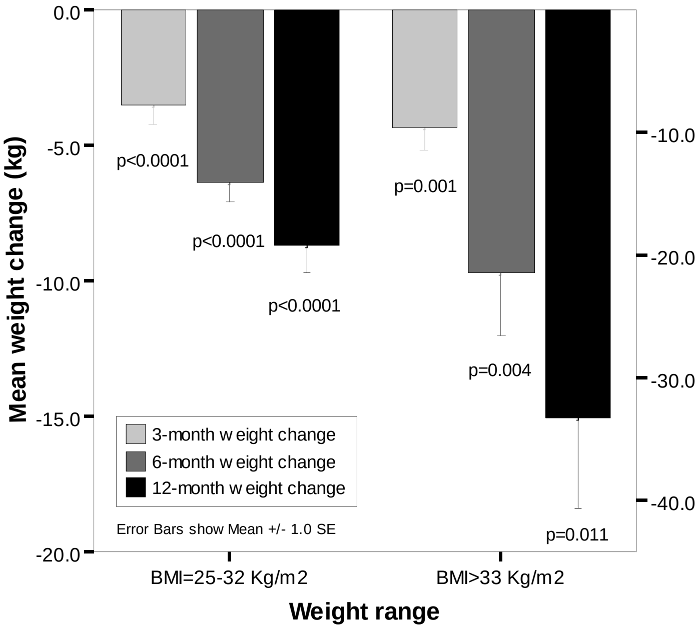

Figure 3. Mean weight changes from baseline at 3-month, 6-month and 12-m Group I and Group II patients. Data are expressed as the mean ± standard er (SE). BMI = body mass index.

The Study Population

The alarming prevalence rates of diabetes and diabetes-related complications and their profound and disparate consequences in African-American and Hispanic women are a subject of universal concern. Collectively, our four study centers have access to a unique distribution of women with high risks of diabetes and The Metabolic Syndrome: The University of Tennessee serves a large community of African-American and Vietnamese women; the Albert Einstein Centers, with affiliated practices located in the Bronx and the Washington Heights section of Manhattan, provide an opportunity to enroll women of several Latina heritages; and The New York Medical College - Westchester Medical Center site currently follows a large, socio-economically diverse, South Asian population that includes many staff health care professionals and their families, as well as Asian-Americans from neighboring communities. Clearly, minority women from all these communities are a critical target for early preventive interventions.

RESEARCH PLAN

Overview of the Study

Investigators at three study sites will conduct a pilot study to improve insulin sensitivity, prevent weight gain, and reduce visceral adiposity in African-American, Hispanic, South Asian, and other high risk populations of normoglycemic, hyperinsulinemic women with midlife weight gain. Enhance the Metabolic Profile of Women with Insulin Resistance (EMPOWIR) will evaluate three treatment protocols in a randomized, double-blind, clinical trial and optional, post-treatment surveillance analysis. The objective of the study is to compare the effect of carbohydrate modified diet alone and in combination with metformin (MF) and Avandamet® (metformin plus rosiglitazone (RSG)) on insulin parameters in hyperinsulinemic, peri- and post-menopausal women with progressive weight gain (Syndrome W) prior to the onset of overt glycemic abnormalities or full-blown Metabolic Syndrome. It will address a wide range of ethnically and economically diverse non-diabetic women (aged 35-55) who meet study inclusion criteria seen at three academic medical centers: The Hispanic Center of Excellence and The Menopause Research and Treatment Center at Albert Einstein College of Medicine/ Montefiore Hospital; The Center for Diabetes and Endocrine Care at New York Medical College/Westchester Medical Center, The Diabetes Center of Excellence at The University of Tennessee and their affiliated practices.

All women will receive the calorie-reduced, carbohydrate modified (sugar restricted, low glycemic index) diet based on large servings of vegetables, fruits, lowfat proteins and dairy products and limited refined carbohydrates and treats. Devised 30 years ago and field-tested in more than a thousand patients in the past decade, this dietary program integrates scientific findings and recommendations from numerous current research studies.

A 3-phase, 3-arm trial will be conducted in a final study population of 75 normoglycemic hyperinsulinemic women (glucose-mediated AUC-insulin >100mU/ml) randomized (Phase 1) to an initial 6-month treatment of study diet, with placebo, metformin, or Avandamet® (rosiglitazone plus metformin). Subjects initially randomized to placebo arm will be reassigned after 6 months to one of the two active treatment arms (Phase 2). In an optional follow-up surveillance study (Phase 3), women attaining goal weight (>10% loss of body weight/12 months), will be re-randomized to placebo or active treatment (with metformin, Avandamet®); a survival analysis (with time to study withdrawal defined as 25% weight regain) will assess the

long-term effect of treatment. (If fewer than 20 patients are available/ eligible for the optional Phase 3, then a single combination arm will be substituted to preserve power estimates.)
Initial funding will also be used to develop a core program to facilitate additional research at EMPOWIR'S two affiliated Clinical Research Centers (CRC's) at the Albert Einstein College of Medicine and The University of Tennessee to include: (a) pre- and post-treatment assessments of appetite and body weight regulation; (b) baseline and follow-up functional imaging of brain and muscle; (c) metabolic studies to evaluate the independent and collective medical and dietary components; (d) epigenetic studies to identify relevant polymorphisms and cohort-based gene-determined environmental interactions.

STUDY OBJECTIVES AND ENDPOINTS

Outcome Measures

The primary outcome variables will be 6-month change in fasting and 1-hour insulin in the placebo *vs.* the combined metformin and avandamet groups.
Secondary outcome measures are 6 and 12 month change from baseline in body weight, DXA-based change in fat compartments and relevant ratios (total abdominal fat compartment at AECOM and WMC sites; CT scan-determined visceral adipose tissue at University of Tennessee); HOMA; total and multimeric adiponectin; and other cardiovascular risk and inflammatory markers, including lipids, c-reactive protein, TNF-α, leptin, ghrelin, and resistin; and IGF-1 and IGF-binding proteins.

Study Overview

(Please see Study Flow Chart on the following page.)
Chart review of patients at the 3 study Centers and their affiliated General Medical and Gynecology practices and public announcements will be used to identify non-diabetic women who meet selection criteria who will be invited to participate in the study. After obtaining consent, and additional screening for suitability, 80 patients will undergo glucose tolerance testing and additional tests to determine if they meet study inclusion and exclusion criteria. Following a 1-month lead-in phase, an initial study population of 75 women from 3 clinical sites will be randomized to placebo (diet and lifestyle modification) or one of two active study treatment arms for a 6-month study (Phase 1); patients in the diet plus placebo group will then be crossed over to one of the two active study arms for a 2nd six months (Phase 2). (A third phase, to assess the effects of medication withdrawal using time to withdrawal as the outcome variable, is presented as an optional Phase 3 extension of the study).

The three arms will use (as detailed in Table 1):

1. placebo metformin and rosiglitazone
2. metformin, initial dose 500 mg BID with titration to 2000mg/day (by adding one 500mg tab weekly on weeks 3 and 4) with placebo rosiglitazone.
3. Avandamet® (administered as rosiglitazone 2 mg BID) plus metformin 500 mg BID (to be titrated to 2000 mg/day.)

A dosage escalation schedule, as outlined, will be utilized to minimize potential gastrointestinal side effects which may accompany the initiation of metformin.

The Study Pharmacist at Westchester Medical Center who oversees randomization in several hundred clinical trials per year will perform the randomization and distribute medications to all study sites.

SUMMARY OF EMPOWIR STUDY

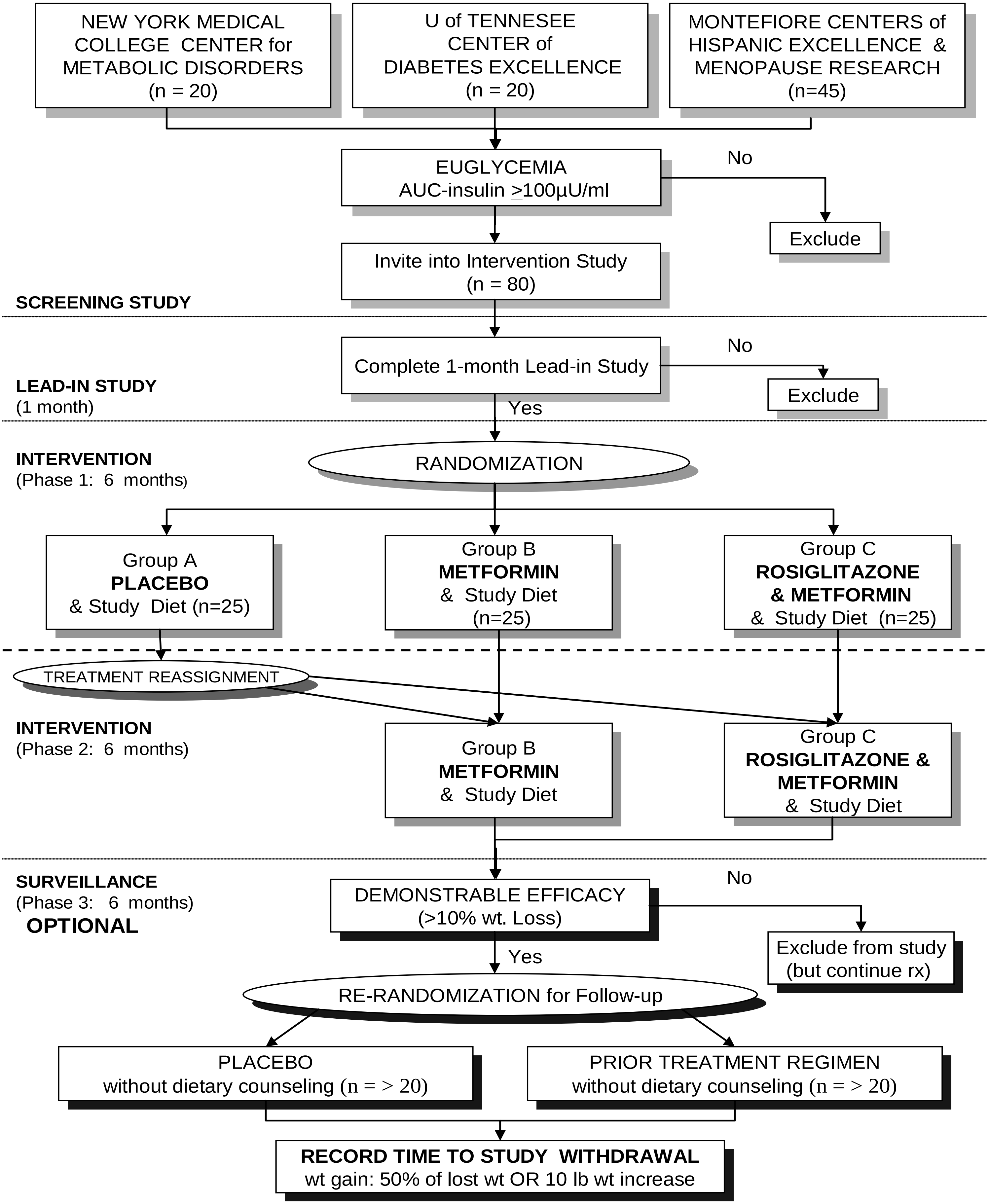

Table 1. Randomization Schedule

	GROUP A<br>(PLACEBO)	GROUP B<br>METFORMIN (MF)<br>***Plus***<br>PLACEBO ROSIGLITAZONE (RSG)	GROUP C<br>METFORMIN (MF)<br>***plus***<br>ROSIGLITAZONE (RSG)
**Week 1**	placebo (RSG) 2 tabs/day<br><br>placebo (MF) 2 tabs/day	placebo (RSG) 2 tabs/ day<br><br>MF 500 mg 1 tab BID	RSG 2 mg 1 tabs BID<br><br>MF 500 mg 1 tabs BID
**Weeks 2 and 3**	placebo (RSG) 1 tab BID<br><br>placebo (MF) 3 tabs/day	placebo (RSG) 1 tab BID<br><br>MF 500 mg 3 tabs/day	RSG 2 mg 1 tab BID<br><br>MF 500 mg 3 tabs/day
**Week 4**	placebo (RSG) 1 tab BID<br><br>placebo (MF) 4 tabs/day	placebo (RSG) 1 tab BID<br><br>MF 500 mg 2 tabs BID	RSG 2 mg 1 tab BID<br><br>MF 500 mg 2 tabs BID
**TOTAL DOSE<br>Weeks 5-26**	2 TABS PLACEBO RSG +<br>4 TABS PLACEBO MF	2 TABS PLACEBO RSG +<br>2000 MG METFORMIN	4 MG ROSIGLITAZONE +<br>2000 MG METFORMIN

Inclusion Criteria

1. Healthy, non-diabetic women with "≥20 pound weight gain since their twenties"
2. Age: 35-55
3. Peri-menopausal (FSH ≥25 on day 2-3 of cycle) or postmenopausal status
4. Body Mass Index (BMI) 25-35 kg/m^2
5. Either:
   (a). a single blood pressure recording ≥135/85 or the use of blood pressure medication OR
   (b) HDL≤50mg/dl or triglycerides ≥150 mg/dl or the use of lipid modifying medication
6. Area-under-the-curve (AUC-)insulin level ≥100μU/ml
   along with normal fasting (≤100 mg/dl) & postprandial ((≤200 mg/dl) glucose determinations following a 75-gram standard oral glucose tolerance test.

Exclusion Criteria

1. known diabetes, fasting blood sugar ≥ 110 mg/dl or
   HbA-1-C≥6.0%
2. known hepatic disease or ALT>40
3. known renal disease or creatinine ≥ 1.4
4. known severe pulmonary disease
5. chronic acidosis of any etiology
6. Congestive heart failure (NYS Category 1), treated or untreated
7. Cancer – active within 5 years
8. current alcoholism or other substance abuse
9. co-morbid psychiatric disorder, which in the opinion of the screening physician would require concomitant psychotherapy as part of obesity management
10. currently untreated thyroid abnormality (TSH≤0.2 or ≥4mIU/L)
11. pregnancy or contemplation of pregnancy
12. use of TZD or metformin within the past year
13. allergy to TZD or biguanide
14. use of FDA approved or alternate obesity agent within 6 months of the study
15. history of pseudotumor cerebri
16. other impairment, such as a history of medication noncompliance, which in the judgment of the screening clinician, would preclude active study participation.

Procedures, Clinical Assessments, Laboratory Assessments, and Safety Monitoring

Weight, BMI, waist and hip circumference, blood pressure will be recorded and appetite ratings, exercise log and food dairies evaluated weekly for the first six weeks and monthly for Phase 1. During the study visits, a symptom check list will be used to inquire about new symptoms and any change in frequency or severity of preexisting (baseline) conditions. Bloods will be drawn for CBC, electrolytes, and comprehensive metabolic profiles at the 1, 2-, 3-, 6- and 12-month study visits (noted in Tables 2 and 3).

GTT and insulin response curves will be obtained at baseline and at the end of 6 and 12 months in study completers.

Adverse events will be monitored and recorded by the study coordinators in accordance with IRB governance and guidelines at the three participating institutions. Serious adverse events (hospitalization, death or pregnancy) will be reported to GSK within 24 hours of obtaining SAE information.

Study Timeline

Projected start date: Sept 1, 2006
Projected study completion date: August 30, 2008 (without optional phase)

Time-table assumptions:
The investigators anticipate a rapid study start-up phase due to:

a. general ease of enrollment of obesity studies
b. investigators' access to large and diverse patient populations in their individual practices and the community
c. availability of field tested patient education materials and study instruments
d. the availability of nursing and nutrition staff with broad experience in the conduct of clinical trials (e.g., The Women's Health Initiative, Diabetes Prevention Trial) in related areas.

These factors will improve the study efficiency and reduce study costs

Table 2. *Study Timetable*

Study Year	**Year one**				**Year two**				**Year three**			
Calendar Year	Sept 2006-August 2007				Sept 2007-August 2008				Sept 2008-August 2009			
Calendar Month	1 – 3	4 – 6	7 – 9	10 – 12	1 – 3	4 – 6	7 – 9	10 – 12	1 – 3	4 – 6	7 – 9	10 – 12
Start-up	*											
Screening &Enrollment	*	*	*									
Lead-in (1 month)	*	*	*	*								
Intervention I (6 months)		*	*	*	*							
Intervention II (6 months)			*	*	*	*	*					
Surveillance (6 months)					*	*	*	*	*			
Data analysis, study report							*	*	*	*	*	*

STATISTICAL ANALYSES AND POWER CALCULATIONS

Assumptions
Three power calculations were performed incorporating the significance levels and sample size of TZD-based interventions in recently reported studies and comparator trials. An initial sample size of 25 per group is a realistic estimate and is based on the following assumptions:

1. Placebo treatment even with intensive dietary intervention (which will not be provided after the lead-in phase of EMPOWIR) has low rates of weight loss and high drop out rates at 6 and 12 months of study follow-up in the majority of published studies
2. data from observational and population based studies show annual weight gain in women in the study age range and BMI category
3. Metformin has a large treatment effect in hyperinsulinemic women and, in combination with hypocaloric carbohydrate modified diet, reduced fasting insulin by 25-40% and 1-hour (peak) insulin by 50% in our preliminary data. (mean body weight change 12.5% at 1-year).
4. Enrollment goals assume 20% drop out during Phase 2
5. **The study is not powered for subgroup analysis by race or menopausal status.** However, depending on the ethnic distribution of the final study population and the relative magnitude of the overall treatment effect, a post-hoc two group – minority (non-white) *vs.* "other" (white) subjects – may be contemplated.

Summary of sample size calculations: (provided by Dept of Epidemiology and Biostatistics, School of Public Health, New York Medical College)

The study design comprises 3 groups in one pre-treatment (Phase 1) and one post-treatment (Phase 3) measurement of outcome. Preliminary data from our open label use of metformin and other randomized clinical trials(24) suggest that metformin affords large treatment effects. Nonetheless, as the large effect may be due in part to placebo, we will structure our sample size to detect more moderate treatment effects. Using an analysis of covariance, a sample size of 60 patients (20 per group) will provide at least 80% power to detect a moderate to large effect size (f=.40) for a two-sided test of significance at a critical value of .05. While 20 patients per group are required to meet the anticipated effects, we will target 25 per group to allow for 20% attrition. The proposed attrition rate is less than rates reported in research studies of other medications evaluated for weight reduction, such as sibutramine (32%) and xenical (24%). However, the rate reflects attrition rates from our own preliminary data and from other published studies of patients treated with metformin analyzed using an intention-to-treat or comparable analysis such as studies of obesity in women, and children, PCOS, and HIV-related lipodystrophy.

Phase 3 is written as an optional add-on to the study. During this period participants will be randomized a second time and a time-to-event (i.e., survival) analysis will be used to compare the placebo to the treated group. The clinical endpoint will be a 50% increase over post-treatment baseline (or a weight gain of 10 pounds, whichever comes first). These are conservative estimates based on our preliminary findings and prior experience in various patient subsets, as well as, other pharmacological studies of weight maintenance in patients who have lost weight.

Statistical Plan

Mean, median, range and distribution of all baseline study variables will be determined (SPSS (13.0); site differences will be assessed using an Analysis of Variance (ANOVA). All primary and secondary outcome variables will be evaluated using an intention-to-treat analysis with a last observation carried forward (LOCF) for missing data, Paired t-tests will be used to calculate baseline changes in insulin parameters, body weight, BMI, cardiovascular risk factors, and related serum markers (adiponectin, leptin, ghrelin, resistin, C-reactive protein and other inflammatory markers, vitamin D and IGF-binding proteins at 6 and 12 months. Non-parametric measures will be used for comparison of group mean changes at 6 and 12 months. Statistical models will be used to calculate the primary (crude, non-adjusted) treatment effect of the respective study arms and for covariate adjustment by age, initial BMI category and menopausal status, and study site.

Reference List

(1) Colditz GA, Willett WC, Rotnitzky A, Manson JE. Weight gain as a risk factor for clinical diabetes mellitus in women. Ann Intern Med 1995 April 1;122(7):481-6.

(2) Manson JE, Colditz GA, Stampfer MJ et al. A prospective study of obesity and risk of coronary heart disease in women. N Engl J Med 1990 March 29;322(13):882-9.

(3) Kannel WB, D'Agostino RB, Cobb JL. Effect of weight on cardiovascular disease. Am J Clin Nutr 1996 March;63(3 Suppl):419S-22S.

(4) Manson JE, Willett WC, Stampfer MJ et al. Body weight and mortality among women. N Engl J Med 1995 September 14;333(11):677-85.

(5) Seidell JC. Societal and personal costs of obesity. Exp Clin Endocrinol Diabetes 1998;106 Suppl 2:7-9.

(6) Kuczmarski RJ, Flegal KM, Campbell SM, Johnson CL. Increasing prevalence of overweight among US adults. The National Health and Nutrition Examination Surveys, 1960 to 1991. JAMA 1994 July 20;272(3):205-11.

(7) Mogul HR, Weinstein BI, Mogul DB et al. Syndrome W: a new model of hyperinsulinemia, hypertension and midlife weight gain in healthy women with normal glucose tolerance. Heart Dis 2002 March;4(2):78-85.

(8) Goldman EL. 'Syndrome W' flags insulin resistance in perimenopause: Abdominal weight gain, white coat hypertension characterize the disorder. Internal Medicine News 1999 Jul 1;1-2.

(9) Dimartino-Nardi J. Premature adrenarche: findings in prepubertal African-American and Caribbean-Hispanic girls. Acta Paediatr Suppl 1999 December;88(433):67-72.

(10) Mogul HR, Peterson SJ, Weinstein BI et al. Metformin and carbohydrate-modified diet, a novel obesity treatment protocol: preliminary findings from a case series of non-diabetic women with midlife weight gain and hyperinsulinemia. Heart Disease 2001 September 1;3(5):285-92.

(11) Mogul HR, Peterson SJ, Weinstein BI et al. Long-term (2-4 year) weight reduction with metformin plus carbohydrate-modified diet in euglycemic, hyperinsulinemic, midlife women (Syndrome W)
14. Heart Dis 2003 November;5(6):384-92.

(12) Purnell JQ, Hokanson JE, Marcovina SM et al. Effect of excessive weight gain with intensive therapy of type 1 diabetes on lipid levels and blood pressure:

results from the DCCT. Diabetes Control and Complications Trial. JAMA 1998 July 8;280(2):140-6.

(13) Turner RC, Cull CA, Frighi V, Holman RR. Glycemic control with diet, sulfonylurea, metformin, or insulin in patients with type 2 diabetes mellitus: progressive requirement for multiple therapies (UKPDS 49). UK Prospective Diabetes Study (UKPDS) Group. JAMA 1999 June 2;281(21):2005-12.

(14) Odeleye OE, de-Courten M, Pettitt DJ, Ravussin E. Fasting hyperinsulinemia is a predictor of increased body weight gain and obesity in Pima Indian children. Diabetes 1997 August;46(8):1341-5.

(15) Folsom AR, Vitelli LL, Lewis CE et al. Is fasting insulin concentration inversely associated with rate of weight gain? Contrasting findings from the CARDIA and ARIC study cohorts. Int J Obes Relat Metab Disord 1998 January;22(1):48-54.

(16) Campbell IW, Menzies DG, Chalmers J et al. One year comparative trial of metformin and glipizide in type 2 diabetes mellitus. Diabete Metab 1994 July;20(4):394-400.

(17) UK Prospective Diabetes Study (UKPDS) Group. Effect of intensive blood-glucose control with metformin on complications in overweight patients with type 2 diabetes (UKPDS 34). UK Prospective Diabetes Study (UKPDS) Group [published erratum appears in Lancet 1998 Nov 7;352(9139):1557]. Lancet 1998 September 12;352(9131):854-65.

(18) Fontbonne A, Charles MA, Juhan V, I et al. The effect of metformin on the metabolic abnormalities associated with upper-body fat distribution. BIGPRO Study Group. Diabetes Care 1996 September;19(9):920-6.

(19) Crave JC, Fimbel S, Lejeune H et al. Effects of diet and metformin administration on sex hormone-binding globulin, androgens, and insulin in hirsute and obese women. J Clin Endocrinol Metab 1995 July;80(7):2057-62.

(20) Casimirri F, Biscotti M, Gambineri A. Metformin improves insulin, body fat distribution, and androgens in obese women with and without the polycystic ovary syndrome (abstract). Int J Obes 1997;(21):S61.

(21) Nestler JE, Jakubowicz DJ. Decreases in ovarian cytochrome P450c17 alpha activity and serum free testosterone after reduction of insulin secretion in polycystic ovary syndrome. N Engl J Med 1996 August 29;335(9):617-23.

(22) Moghetti P, Castello R, Negri C et al. Metformin effects on clinical features, endocrine and metabolic profiles, and insulin sensitivity in polycystic ovary syndrome: a randomized, double -blind, placebo-controlled 6-month trial, followed by open, long-term clinical evaluation. J Clin Endocrinol Metab 2000 January;85(1):139-46.

(23) Hadigan C, Corcoran C, Basgoz N et al. Metformin in the Treatment of HIV Lipodystrophy Syndrome: A Ramdomized Controlled Trial. JAMA 2000 July 26;284(4):472-7.

(24) Freemark M, Bursey D. The effects of metformin on Body Mass Index and glucose tolerance in obese adolescents with fasting hyperinsulinemia and a family history of type 2 diabetes. Pediatrics 2001;107(4):e55.

(25) Paolisso G, Amato L, Eccellente R et al. Effect of metformin on food intake in obese subjects
137. Eur J Clin Invest 1998 June;28(6):441-6.

(26) Lee A, Morley JE. Metformin decreases food consumption and induces weight loss in subjects with obesity with type II non-insulin-dependent diabetes
147. Obes Res 1998 January;6(1):47-53.

(27) Boden G, Chen X, Ruiz J et al. Mechanisms of fatty acid-induced inhibition of glucose uptake. J Clin Invest 1994 June;93(6):2438-46.

(28) Abbasi F, Kamath V, Rizvi AA et al. Results of a placebo-controlled study of the metabolic effects of the addition of metformin to sulfonylurea-treated patients. Evidence for a central role of adipose tissue. Diabetes Care 1997 December;20(12):1863-9.

(29) Haas DA, Carr BR, Attia GR. Effects of metformin on body mass index, menstrual cyclicity, and ovulation induction in women with polycystic ovary syndrome. Fertil Steril 2003 March;79(3):469-81.

(30) Lee ZSK, Chan JCN, Yeung VTF et al. Plasma insulin, growth hormone, cortisol, and central obesity among young Chinese Type 2 diabetic patients. Diabetes Care 1999 September 1;22(9):1450-7.

(31) Boden G, Chen X, Iqbal N. Acute lowering of plasma fatty acids lowers basal insulin secretion in diabetic and nondiabetic subjects. Diabetes 1998 October;47(10):1609-12.

(32) Boden G, Chen X. Effects of fatty acids and ketone bodies on basal insulin secretion in type 2 diabetes. Diabetes 1999 March;48(3):577-83.

(33) Davidson MB, Peters AL. An overview of metformin in the treatment of type 2 diabetes mellitus. Am J Med 1997 January;102(1):99-110.

(34) Giugliano D, De-Rosa N, Di-Maro G et al. Metformin improves glucose, lipid metabolism, and reduces blood pressure in hypertensive, obese women. Diabetes Care 1993 October;16(10):1387-90.

(35) Charles MA, Morange P, Eschwege E et al. Effect of weight change and metformin on fibrinolysis and the von Willebrand factor in obese nondiabetic

subjects: the BIGPRO1 Study. Biguanides and the Prevention of the Risk of Obesity. Diabetes Care 1998 November;21(11):1967-72.

(36) Ferrannini E. Rosiglitazone and lipid metabolism
281. Diabetologia 2005 January;48(1):6-7.

(37) Negro R, Mangieri T, Dazzi D et al. Rosiglitazone effects on blood pressure and metabolic parameters in nondipper diabetic patients
109. Diabetes Res Clin Pract 2005 October;70(1):20-5.

(38) Meisner F, Walcher D, Gizard F et al. Effect of Rosiglitazone Treatment on Plaque Inflammation and Collagen Content in Nondiabetic Patients. Data From a Randomized Placebo-Controlled Trial
333. Arterioscler Thromb Vasc Biol 2006 January 12.

(39) Wang G, Wei J, Guan Y et al. Peroxisome proliferator-activated receptor-gamma agonist rosiglitazone reduces clinical inflammatory responses in type 2 diabetes with coronary artery disease after coronary angioplasty
192. Metabolism 2005 May;54(5):590-7.

(40) Baillargeon JP, Jakubowicz DJ, Iuorno MJ et al. Effects of metformin and rosiglitazone, alone and in combination, in nonobese women with polycystic ovary syndrome and normal indices of insulin sensitivity
329. Fertil Steril 2004 October;82(4):893-902.

(41) Lv L, Liu Y. Effect of rosiglitazone on endocrine, metabolism and ovulatory performance in patients with polycystic ovary syndrome and insulin resistance
272. J Huazhong Univ Sci Technolog Med Sci 2004;24(5):480-2.

(42) Yilmaz M, Bukan N, Ayvaz G et al. The effects of rosiglitazone and metformin on oxidative stress and homocysteine levels in lean patients with polycystic ovary syndrome
114. Hum Reprod 2005 December;20(12):3333-40.

(43) Dereli D, Dereli T, Bayraktar F et al. Endocrine and metabolic effects of rosiglitazone in non-obese women with polycystic ovary disease
148. Endocr J 2005 June;52(3):299-308.

(44) Summaries for patients. Rosiglitazone treatment for fat redistribution and metabolic abnormalities caused by anti-HIV therapy
399. Ann Intern Med 2004 May 18;140(10):I36.

(45) Summaries for patients. HIV lipodystrophy: rosiglitazone or metformin?
105. Ann Intern Med 2005 September 6;143(5):I38.

(46) van Wijk JP, de Koning EJ, Cabezas MC et al. Comparison of rosiglitazone and metformin for treating HIV lipodystrophy: a randomized trial
104. Ann Intern Med 2005 September 6;143(5):337-46.

(47) Kim SH, Abbasi F, Chu JW et al. Rosiglitazone reduces glucose-stimulated insulin secretion rate and increases insulin clearance in nondiabetic, insulin-resistant individuals
344. Diabetes 2005 August;54(8):2447-52.

(48) Mitkov M, Pehlivanov B, Terzieva D. Metformin versus rosiglitazone in the treatment of polycystic ovary syndrome
334. Eur J Obstet Gynecol Reprod Biol 2005 December 14.

(49) Gerstein HC, Yusuf S, Holman R et al. Rationale, design and recruitment characteristics of a large, simple international trial of diabetes prevention: the DREAM trial
1. Diabetologia 2004 September;47(9):1519-27.
