# Supplementary material for: Carbohydrate Modified Diet & Insulin Sensitizers Reduce Body Weight & Modulate Metabolic Syndrome Measures in EMPOWIR (Enhance the Metabolic Profile of Women with Insulin Resistance): A Randomized Trial of Normoglycemic Women with Midlife Weight Gain
Source: PLoS One. 2014 Sep 26;9(9):e108264. doi: 10.1371/journal.pone.0108264 (PMC4178125; doi:10.1371/journal.pone.0108264)
Supplement: Protocol S2 — Trial protocol as submitted to New York Medical College IRB. (PDF) [file pone.0108264.s008.pdf]

GlaxoSmithKline
Clinical Development & Medical Affairs, North America

information:

Name of Investigator: Harriette R. Mogul MD MPH

Name of Institution: New York Medical College

Address of Institution: Department of Medicine, Division of Endocrinology
New York Medical College
Valhalla, NY 10595

Phone: 914-594-4245 FAX: 914-594-3340

Email: hrmogul@NYMC.edu

Title of Proposal:

EMPOWIR: ENHANCE the METABOLIC PROFILE OF WOMEN WITH INSULIN RESISTANCE

SCIENTIFIC RATIONALE FOR STUDY/ SCIENTIFIC STUDY OBJECTIVES:

Background and Study Rationale

Progressive weight gain that starts in the fourth and fifth decades is commonly reported by women from all ethnic and socio-economic groups. Our previous data suggest that, in large and diverse subpopulations of healthy-appearing women this midlife weight gain may represent the earliest clinical manifestation of insulin resistance – demarcated by increased insulin response curves in the presence of completely normal glucose tolerance tests. We termed the disorder Syndrome W to highlight its defining triad of **w**eight gain, **w**aist gain and **w**hite-coat hypertension in **w**omen and its role as an alphabetic and chronologic antecedent to the better known Syndrome X. As in other disorders of insulin action in younger women, including Polycystic Ovarian Syndrome (PCOS), early adrenarche, and precocious puberty, Syndrome W is, presumably, a harbinger of The Metabolic Syndrome and Type 2 diabetes at an early and optimal period for intervention.

Preliminary data from our first pilot study suggested that metformin, in combination with a hypocaloric, low-fat, carbohydrate modified dietary program produced significant and sustainable weight loss in women with Syndrome W, with notable reductions in fasting insulin levels. These findings supported hypotheses that insulin elevation might be an antecedent, as well as a consequence, of weight gain, accounting for a progressive and intractable weight spiral as women transition from their forties to their sixties. Additional two to four year follow-up in an intention-to-treat analysis of consecutive women who lost ≥10% of their body weight after one year of the treatment regimen further suggests that this composite intervention prevents weight regain and the onset of overt glucose impairment. The protocol evolved from evaluation and treatment of several hundred patients seen in The Endocrine Faculty Practice, and The Obesity Research and Treatment Center at Westchester Medical Center over a ten year period and has been highly successful in a broad ethnic range of normoglycemic, hyperinsulinemic subjects. These include midlife women with weight gain; younger overweight-obese women with Polycystic Ovarian Syndrome; overweight men with upper body obesity and obese adolescents – populations which have not been comparably treated in prior studies which focus predominantly on subjects with discernible glycemic abnormalities. The magnitude and duration of the treatment effect and its potential impact in the context of the current obesity and diabetes epidemics suggest that more rigorous study should be undertaken with a randomized clinical trial.

PPAR agonists including thiazolidinediones (TZD's) are a newer category of insulin sensitizers with increasingly wide and well-studied positive attributes, including redistribution of fat depots, increased adiponectin secretion, and reduction of inflammatory and proinflammatory markers. Clinical use of TZD's and metformin has been expanded to encompass additional disorders of insulin resistance, including PCOS, non-alcoholic hepatitis (NASH), and HAART (highly active antiretroviral treatment) – induced HIV lipodystrophy.

The combination of metformin and rosiglitazone (Avandamet®) is FDA-approved for the treatment of hyperglycemia in patients with Type 2 diabetes. Previous clinical research and recent laboratory data – metformin mediated reduction of hepatic LKB1in murine

knockout models (Science, Nov, 2005) suggest that the two categories of insulin sensitizers have independent and additive mechanisms of action that could target and, ultimately, modulate the underlying pathogenesis of insulin resistance.

Comparison studies suggest that TZD’s may have a greater insulin sensitizing action and provide greater reduction in hyperinsulinemia than metformin. However, due to increased adipocyte expression (and possible other mechanisms), weight gain is a common and undesirable side effect of TZD treatment. The addition of metformin to rosiglitazone, along with dietary strategies that reduce endogenous insulin production could prove an ideal therapeutic option to attenuate insulin resistance and preserve ß-cell function in high risk individuals. Early initiation of this dual regimen in normoglycemic subjects with documented hyperinsulinemia could have profound implications for Syndrome W women and for an additional 25% of the adult US population estimated to have other manifestations of The Metabolic Syndrome.

The proposed study differs in several respects from previously published and ongoing clinical trials of TZD’s, metformin, or lifestyle intervention. The primary study question addressed is whether dual treatment regimens which modulate insulin action can reduce hyperinsulinemia and insulin resistance in high risk, but healthy-appearing normoglycemic, hyperinsulinemic subjects identified because of progressive, intractable, midlife weight gain. In contrast to other trials of thiazolidinediones, such as DREAM, ACCORD, BARI-2D, and PIPOD, which enroll patients with overt glycemic abnormalities, EMPOWIR specifically targets normoglycemic women from minority populations and assesses insulin rather than glucose parameters as the primary outcome measures.

Summary of Study Hypotheses

These related hypotheses are derived from clinical observations and emerging research on the role of insulin in the pathogenesis of obesity and related adverse health outcomes.

H_o: Compensatory hyperinsulinemia (independently or in association with abnormalities in insulin action, free fatty acid metabolism or other metabolic correlates) represents a common, generally undetected metabolic abnormality that promotes and sustains obesity in distinct subpopulations.

H_o: Even in the absence of overt glycemic disturbance, insulin elevations should be a primary treatment target of insulin sensitizing agents (along with other synergistic therapies that attenuate insulin resistance and reduce compensatory insulin secretion) in patients at risk for Type 2 diabetes and The Metabolic Syndrome.

H_o: Early identification of hyperinsulinemia and early initiation of insulin sensitizing agents could alter pathogenesis and disease progression in normoglycemic women (Syndrome W) and provide an important treatment model for additional populations at risk for Type 2 diabetes and Metabolic Syndrome.

The Study Population

The alarming prevalence rates of diabetes and diabetes-related complications and their profound and disparate consequences in African-American and Hispanic women are a subject of universal concern. Collectively, our four study centers have access to a unique distribution of women with high risks of diabetes and The Metabolic Syndrome: The University of Tennessee serves a large community of African-American and Vietnamese women; the Albert Einstein Centers, with affiliated practices located in the Bronx and the Washington Heights section of Manhattan, provide an opportunity to enroll women of several Latina heritages; and The New York Medical College - Westchester Medical Center site currently follows a large, socio-economically diverse, South Asian population that includes many staff health care professionals and their families, as well as Asian-Americans from neighboring communities. Clearly, minority women from all these communities are a critical target for early preventive interventions.

Objective and Overview of the Study

to conduct a pilot study to improve insulin sensitivity, prevent weight gain and reduce visceral adiposity in African-American, Hispanic, South Asian, and other high risk populations of normoglycemic, hyperinsulinemic women with midlife weight gain. Enhance the Metabolic Profile of Women with Insulin Resistance (EMPOWIR) will compare three treatment protocols in a randomized, double-blind, clinical trial and optional, post-treatment surveillance analysis.

The objective of the study is to compare the effect of carbohydrate modified diet alone and in combination with metformin (MF) and Avandamet® (metformin plus rosiglitazone (RSG)) on insulin parameters in a wide range of ethnically and economically diverse non-diabetic women (aged 35-55) who meet study selection criteria seen at three academic medical centers: The Hispanic Center of Excellence and The Menopause Research and Treatment Center at Albert Einstein College of Medicine/ Montefiore Hospital; The Diabetes Center of Excellence at The University of Tennessee; and The Center for Diabetes and Endocrine Care at New York Medical College/Westchester Medical Center and their affiliated practices. All women will receive the calorie-reduced, carbohydrate modified (sugar restricted, low glycemic index) diet based on large servings of vegetables, fruits, lowfat proteins and dairy products and limited refined carbohydrates and treats. Devised 30 years ago and field-tested in more than a thousand patients in the past decade, this dietary program integrates scientific findings and recommendations from numerous current research studies.

A 3-arm trial will be conducted in a final study population of 75 normoglycemic hyperinsulinemic women (glucose-mediated AUC-insulin >100mU/ml) randomized (Phase 1) to an initial 6-month treatment of study diet, with placebo, metformin, or Avandamet®. Subjects initially randomized to placebo arm will be reassigned after 6 months to one of the two active treatment arms (Phase 2).

Initial funding will be used to develop a core program to facilitate additional research at

New York Medical College and EMPOWIR'S affiliated Clinical Research Center (CRC) at the Albert Einstein College of Medicine and The University of Tennessee's Diabetes Center to include: (a) pre- and post-treatment assessments of appetite and body weight regulation; (b) baseline and follow-up functional imaging of brain and muscle; (c) metabolic studies to evaluate the independent and collective medical and dietary components; (d) epigenetic studies to identify relevant polymorphisms and cohort-based gene-determined environmental interactions.

Methods:

Study Design	***Overview***<br>Please see attached Study Flow Chart<br>Chart review of patients at the 3 study Centers and their affiliated General Medical and Gynecology practices and public announcements will be used to identify non-diabetic women who meet selection criteria who will be invited to participate in the study. After obtaining consent, and additional screening for suitability, 80 patients will undergo glucose tolerance testing and additional tests to determine if they meet study inclusion and exclusion criteria. Following a 1-month lead-in phase, an initial study population of 75 women from 3 clinical sites will be randomized to placebo (diet and lifestyle modification) or one of two active study treatment arms for a 6-month study (Phase 1); patients in the diet plus placebo group will then be crossed over to one of the two active study arms for a 2nd six months (Phase 2).
Inclusion / Exclusion	**Inclusion Criteria**<br>1. Healthy, non-diabetic women with "≥20 pound weight gain since their twenties"<br>2. Age: 35-55<br>3. Peri-menopausal (FSH ≥25 on day 2-3 of cycle) or postmenopausal status<br>4. Body Mass Index (BMI) 25-35 kg/m^2<br>5. Either:<br>(a). a single blood pressure recording ≥135/85 or the use of blood pressure medication OR<br>6. (b) HDL≤50mg/dl or triglycerides ≥150 mg/dl or the use of lipid modifying medication<br>7. Area-under-the-curve (AUC-)insulin level ≥100µU/ml along with normal fasting (≤100 mg/dl) & postprandial ((≤200 mg/dl) glucose determinations following a 75-gram standard oral glucose tolerance test.<br><br>**Exclusion Criteria**

	1. known diabetes, fasting blood sugar ≥ 110 mg/dl or HbA-1-C≥6.0%<br>2. known hepatic disease or ALT>40<br>3. known renal disease or creatinine ≥ 1.4<br>4. known severe pulmonary disease<br>5. chronic acidosis of any etiology<br>6. Congestive heart failure (NYS Category 1), treated or untreated<br>7. a history of diagnosed or suspected coronary artery disease<br>8. Cancer – active within 5 years<br>9. current alcoholism or other substance abuse<br>10. co-morbid psychiatric disorder, which in the opinion of the screening physician would require concomitant psychotherapy as part of obesity management<br>11. currently untreated thyroid abnormality (TSH≤0.2 or ≥4mIU/L)<br>12. pregnancy or contemplation of pregnancy<br>13. use of TZD or metformin within the past year<br>14. allergy to TZD or biguanide<br>15. use of FDA approved or alternate obesity agent within 6 months of the study<br>16. history of pseudotumor cerebri<br>17. other impairment, such as a history of medication noncompliance, which in the judgment of the screening clinician, would preclude active study participation.

Number of Patients	Total enrollment 75-85 (approx. 20 per site) to capture 60 patients completing the study
Projected Enrollment Timeline (proposed start date, proposed finish date, anticipated enrollment rate)	**Please see attached timeline**<br>Projected start date: Sept 1, 2006<br>Projected study completion date: August 30, 2008 (without optional phase)<br><br>**Time-table assumptions**:<br>The investigators anticipate a rapid study start-up phase due to:<br><br>a. general ease of enrollment of obesity studies<br>b. investigators' access to large and diverse patient populations in their individual practices and the community<br>c. availability of field tested patient education materials and study instruments<br>d. the availability of nursing and nutrition staff with broad

	experience in the conduct of clinical trials (e.g., The Women's Health Initiative, Diabetes Prevention Trial) in related areas.<br><br>These factors will improve the study efficiency and reduce study costs.
Drug Dose<br>(doses, dosing, frequency, duration)	The three arms will use:<br>1. placebo Avandamet® (using the dosage schedule for placebo metformin in the metformin arms below).<br>2. Metformin, initial dose 500 mg BID with titration to 2000mg/day (by adding one 500mg tab weekly on weeks 3 and 4).<br>3. Avandamet 2 mg BID plus metformin 500 mg BID (to be titrated to 2000 mg/day.)<br>The study can also be done with a dual placebo regimen, i.e., substitution of a placebo tablet for both metformin and rosiglitazone, such that placebo and metformin arms receive placebo RSG and the Avandamet arm receives separate tabs for MF and RSG. A detailed table with an initial weekly dosage schedule for all medications in the 3 arms for the lead-in and subsequent phases can be provided on request.<br><br>The Study Pharmacist at Westchester Medical Center who oversees randomization in several hundred clinical trials per year will perform the randomization and distribute medication to all sites.
Study Endpoints/<br>Objectives	The study objective is to compare the effects of metformin, and Avandamet® on markers of insulin resistance – fasting, peak (1-hour) and AUC-insulin, and HOMA-R, and body fat distribution in hyperinsulinemic, peri- and post-menopausal women with progressive weight gain (Syndrome W) prior to the onset of overt glycemic abnormalities or full-blown Metabolic Syndrome.<br><br>**The primary outcome variables** will be 1-year change in fasting and 1-hour insulin (as noted below.)

Data Analysis:

Study Variables<br>(Clinical procedures, laboratory tests, safety assessments)	Weight, BMI, waist and hip circumference, blood pressure will be recorded and appetite ratings, exercise log and food dairies evaluated weekly for the first six weeks and monthly for Phase 1. During the study visits, a symptom check list will be used to inquire about new symptoms and any change in frequency or severity of preexisting (baseline) conditions. Bloods will be drawn for CBC, electrolytes, and comprehensive metabolic

	profiles at the 1, 2-, 3-, 6- and 12-month study visits (noted in Table 1).<br><br>GTT and insulin response curves will be obtained at baseline and at the end of 6 and 12 months in study completers.<br><br>Adverse events will be monitored and recorded by the study coordinators in accordance with IRB governance and guidelines at the three participating institutions. Serious adverse events (hospitalization, death or pregnancy) will be reported to GSK within 24 hours of obtaining SAE information.
Outcome Parameters / Analytical Endpoints	**The primary outcome variables** are change from baseline in fasting and one-hour insulin at 1 year. **Secondary outcome measures** are 6 and 12 month change from baseline in body weight, CT scan-determined **visceral adipose tissue** other fat compartments, and relevant ratio's; total and multimeric adiponectin; and **other cardiovascular risk and inflammatory markers**, including lipids, c-reactive protein, TNF-α, leptin, ghrelin, and resistin; and IGF-1 and IGF-binding proteins.
Statistical Analysis Plan	**Power Calculations and Assumptions**<br>Three power calculations were performed incorporating the significance levels and sample size of TZD-based interventions in recently reported studies and comparator trials. A initial sample size of 25 per group is a realistic estimate and is based on the following assumptions:<br><br>1. Placebo treatment even with intensive dietary intervention (which will not be provided after the lead-in phase of EMPOWIR) has low rates of weight loss and high drop out rates at 6 and 12 months of study follow-up in the majority of published studies<br>2. data from observational and population based studies show annual weight gain in women in the study age range and BMI category<br>3. Metformin has a large treatment effect in hyperinsulinemic women and, in combination with hypocaloric carbohydrate modified diet, reduced fasting insulin by 25-40% and 1-hour (peak) insulin by 50% in our preliminary data. (with a mean body weight change 12.5% at 1-year).<br>4. Enrollment goals assume 10% drop out during Phase 2

5. **The study is not powered for subgroup analysis by race or menopausal status.** However, depending on the ethnic distribution of the final study population and the relative magnitude of the overall treatment effect, a post-hoc two group – minority (non-white) *vs.* "other" (white) subjects – may be contemplated.

Summary of sample size calculations:
The study design comprises 3 groups in one pre-treatment (Phase 1) and one post-treatment (Phase 3) measurement of outcome. Preliminary data from our open label use of metformin and other randomized clinical trials suggest that metformin affords large treatment effects. Nonetheless, as the large effect may be due in part to placebo, we will structure our sample size to detect more moderate treatment effects. Using an analysis of covariance, a sample size of 60 patients (20 per group) will provide at least 80% power to detect a moderate to large effect size (*f*=.38) for a two-sided test of significance at a critical value of .05. While 20 patients per group are required to meet the anticipated effects, we will target 25 per group to allow for 20% attrition. The proposed attrition rate is less than rates reported in research studies of other medications evaluated for weight reduction, such as sibutramine (32%) and xenical (24%). However, the rate reflects attrition rates from our own preliminary data and from other published studies of patients treated with metformin analyzed using an intention-to-treat or comparable analysis such as studies of obesity in women, and children, PCOS, and HIV-related lipodystrophy.

Statistical Plan

Mean, median, range and distribution of all baseline study variables will be determined (SPSS (13.0); site differences will be assessed using an Analysis of Variance (ANOVA). All primary and secondary outcome variables will be evaluated using an intention-to-treat analysis with a last observation carried forward (LOCF) for missing data, Paired t-tests will be used to calculate baseline changes in insulin parameters, body weight, BMI, cardiovascular risk factors, and related serum markers (adiponectin, leptin, ghrelin, resistin, C-reactive protein and other inflammatory markers, vitamin D and IGF-binding proteins at 6 and 12 months. Non-parametric measures will be used for comparison of group mean changes at 6 and 12 months. Statistical models will be used to calculate the primary (crude, non-adjusted) treatment effect of the respective study arms and for covariate adjustment by age, initial BMI category and menopausal status,

	and study site.

Publication / Presentation Plan:

Following the analysis of baseline data, abstracts will be submitted for presentation at annual meetings, including The Endocrine Society, The ADA, The American College of Cardiology, NAASO (The National Association for The Advancement of the Study of Obesity, NAMS, (The North American Society for Menopause). Publications will be prepared and submitted at regular intervals, following the collation of baseline data, and the completion of each study phase. A final summary manuscript will be submitted within 6 months of the study completion.

Estimated Study Budget/Requested Support (per patient cost, study medication, other supplies):

Please see attached

Funding requested from any other source? No:__√_Yes:____If yes, give details:

Additional funding from Public Health sectors will be requested after initiation of EMPOWIR.

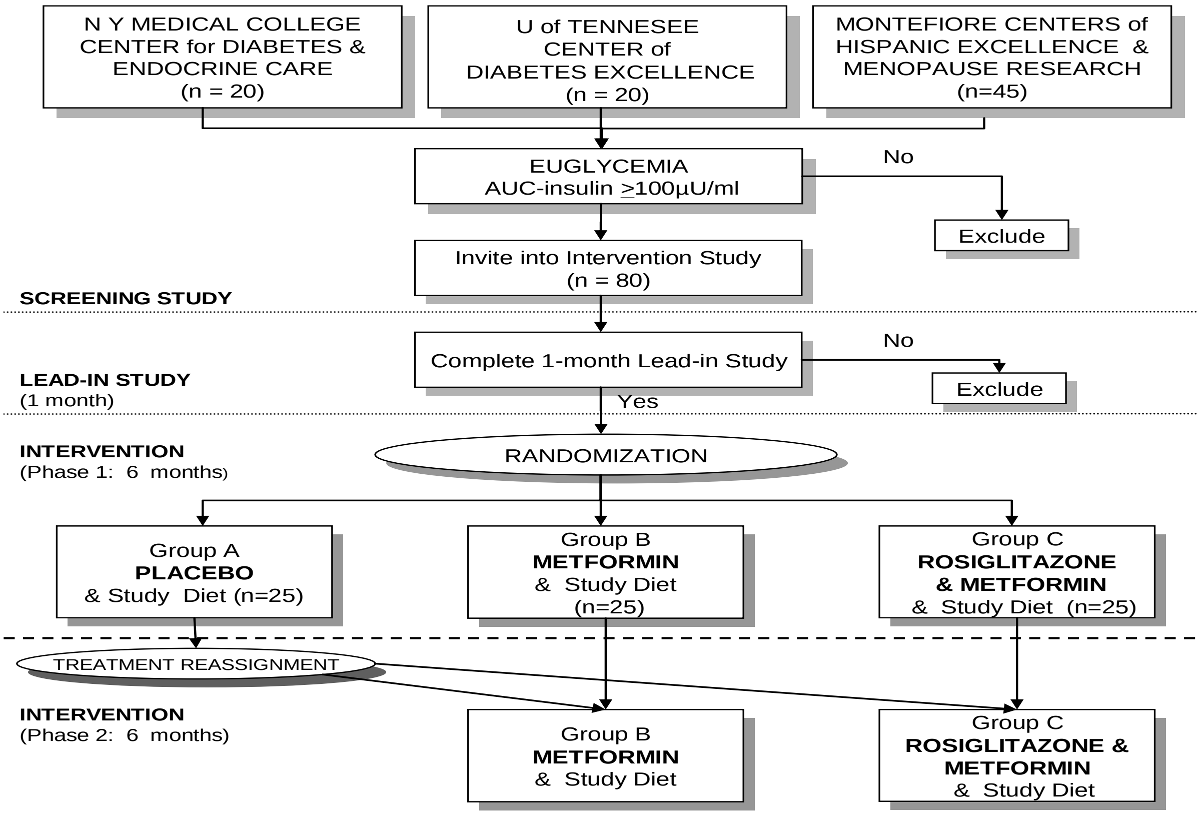

SUMMARY OF EMPOWIR:

ENHANCE THE METABOLIC PROFILE OF WOMEN WITH INSULIN RESISTANCE

A Rosiglitazone Unsolicited Study Proposal

GlaxoSmithKline
